# Notch activation shifts the fate decision of senescent progenitors toward myofibrogenesis in human adipose tissue

**DOI:** 10.1111/acel.13776

**Published:** 2023-01-08

**Authors:** Nathalie Boulet, Anaïs Briot, Valentin Jargaud, David Estève, Anne Rémaury, Chloé Belles, Pénélope Viana, Jessica Fontaine, Lucie Murphy, Catherine Déon, Marie Guillemot, Catherine Pech, Yaligara Veeranagouda, Michel Didier, Pauline Decaunes, Etienne Mouisel, Christian Carpéné, Jason S. Iacovoni, Alexia Zakaroff‐Girard, Jean‐Louis Grolleau, Jean Galitzky, Séverine Ledoux, Jean‐Claude Guillemot, Anne Bouloumié

**Affiliations:** ^1^ Metabolic and Cardiovascular Research Institute, Team Dinamix INSERM UMR1297 and Université de Toulouse Toulouse France; ^2^ Sanofi, Research & Development Translational Sciences, Biochemistry Team Chilly‐Mazarin cedex France; ^3^ Sanofi, Research & Development Exploratory Unit, Proteomic Team Toulouse France; ^4^ Metabolic and Cardiovascular Research Institute, Team MetaDiab INSERM UMR1297 and Université de Toulouse Toulouse France; ^5^ Metabolic and Cardiovascular Research Institute, Bioinformatic Core Facility INSERM UMR1297 and Université de Toulouse Toulouse France; ^6^ Plastic, reconstructive and aesthetic surgery CHU Toulouse Rangueil Toulouse France; ^7^ Center of Obesity, Explorations fonctionnelles, Louis Mourier Hospital (APHP) Université Paris Diderot Colombes France

**Keywords:** adipogenesis, adipose tissue, myofibrogenesis, NOTCH signaling, obesity, progenitors, senescence

## Abstract

Senescence is a key event in the impairment of adipose tissue (AT) function with obesity and aging but the underlying molecular and cellular players remain to be fully defined, particularly with respect to the human AT progenitors. We have found distinct profiles of senescent progenitors based on AT location between stroma from visceral versus subcutaneous AT. In addition to flow cytometry, we characterized the location differences with transcriptomic and proteomic approaches, uncovering the genes and developmental pathways that are underlying replicative senescence. We identified key components to include INBHA as well as SFRP4 and GREM1, antagonists for the WNT and BMP pathways, in the senescence‐associated secretory phenotype and NOTCH3 in the senescence‐associated intrinsic phenotype. Notch activation in AT progenitors inhibits adipogenesis and promotes myofibrogenesis independently of TGFβ. In addition, we demonstrate that NOTCH3 is enriched in the premyofibroblast progenitor subset, which preferentially accumulates in the visceral AT of patients with an early obesity trajectory. Herein, we reveal that NOTCH3 plays a role in the balance of progenitor fate determination preferring myofibrogenesis at the expense of adipogenesis. Progenitor NOTCH3 may constitute a tool to monitor replicative senescence and to limit AT dysfunction in obesity and aging.

## Graphical abstract

Obese individuals accumulate senescent progenitors preferentially in visceral adipose tissue (AT). Replicative senescence of AT progenitors increases NOTCH3 expression. Notch activation enhances SFRP4 and INHBA in the senescence‐associated secretory phenotype and promotes myofibrogenic fate while inhibiting adipogenesis.

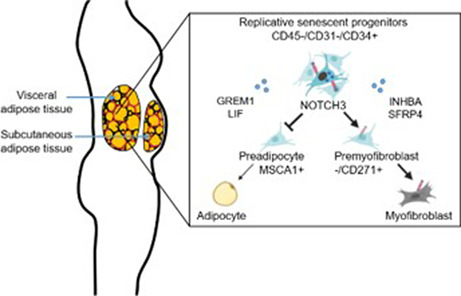

## 1 INTRODUCTION

Central obesity and aging share similarities in terms of their metabolic and inflammatory alterations, such as the accumulation of visceral fat depots, insulin resistance, chronic low‐grade inflammatory state (Trim et al., 2018), and elevated risk to develop severe chronic pathologies, including type 2 diabetes and cardiovascular diseases. The fact that obesity shares features with aging led to the concept that excessive fat mass accelerates the onset of aging‐related disorders (Burton & Faragher, 2018). Cellular senescence, known to be involved in both aging and age‐related disorders (van Deursen, 2014), has recently also been found to play a role in obesity‐associated pathologies (Palmer, Gustafson, et al., 2019). While senescent cells can be protective against cancer development (Campisi, 2013), their accumulation in metabolically active organs, such as the liver (Ogrodnik et al., 2017) and adipose tissue (AT; Minamino et al., 2009), shows negative effects with aging and obesity. Cell cycle arrest triggered by external or internal stresses including telomere shortening (Shay, 2016), DNA damage, oxidative stress, and the excess of metabolic substrates including ATP (Pini et al., 2021) through the activation of p16Ink4a‐ and p53‐dependent pathways, together with the senescence‐associated beta‐galactosidase (SA‐β‐gal) activity, are hallmarks of AT senescent stroma‐vascular cells. In addition, mature adipocytes also express a form of cell cycle‐independent senescence (Ishaq et al., 2022). The causal role of senescence on impaired energy homeostasis is demonstrated by targeting senescent cells with drug‐inducible “suicide” genes driven by the p16(Ink4a; Palmer, Xu, et al., 2019) or p21 (Wang et al., 2022) promoter or by senolytic drugs in obese and aged mice, strategies associated with reduction of age‐ and obesity‐related metabolic abnormalities. Many of the detrimental effects of senescence are thought to be mediated by the senescence‐associated secretory phenotype (SASP) that affects the healthy neighbor cells by promoting low‐grade inflammation and fibrosis. SASP can spread senescence itself, leading to marked alterations in tissue homeostasis and function and thereby lead to systemic insulin resistance (Xu, Tchkonia, et al., 2015). Whether cell‐intrinsic mechanisms are also involved in this process remains to be established.

Progenitors, identified as CD45^−^/CD34^+^/CD31^−^ stromal cells, are major contributors in the maintenance of AT homeostasis through the renewal of dysfunctional hypertrophic adipocytes and extracellular matrix. Progenitor cells are also important in the regulation of AT growth by providing additional adipocytes leading to AT hyperplasia. Progenitors constitute a heterogeneous mesenchymal stromal cell population of specialized subsets as shown by single‐cell RNA sequencing approaches (Emont et al., 2022). Using single‐cell flow cytometry approaches, we previously identified tissue nonspecific alkaline phosphatase (*ALPL* or MSCA1) and *NGFR* (or CD271) as respective markers of the preadipocyte and the premyofibroblast in human fat depots (Esteve et al., 2019). Impaired adipogenesis has been associated with senescence in aged/obese mice and in human AT stromal cells (Le Pelletier et al., 2021; Xu, Palmer, et al., 2015). Several data are in line with an environmental impact of the SASP‐derived factors including antiadipogenic Activin A (Xu, Palmer, et al., 2015) and pro‐fibrotic Osteopontin (Sawaki et al., 2018). But integration of senescent environmental cues, together with progenitor intrinsic mechanisms controlling their fate, have yet to be defined. In addition, a better understanding of the molecular basis of progenitor aging is of interest to monitor and target senescence of mesenchymal stromal cells for future cell‐based therapy in regenerative medicine.

The anatomical repartition of fat depots and the maintenance of AT expandability are major determinants of human metabolic health. Accumulation of visceral AT (VsAT), as observed in central obesity and with aging, is deleterious and associated with increased risk to develop cardiometabolic pathologies. In the present study, we performed flow cytometry analyses to investigate the stromal senescent state according to fat depot location using a human cohort of obese subjects and unbiased large‐scale transcriptomic and proteomic approaches on human immuno‐selected CD45^−^/CD34^+^/CD31^−^ AT progenitors. Our observations show that TGFbeta/BMP, WNT, and NOTCH developmental pathways are dysregulated in these senescent progenitors. GREM1 and SFRP4, endogenous antagonists to the BMP and WNT pathways, and INHBA were found to be preferentially upregulated in SASP from VsAT. Cells forced into replicative senescence upregulated intrinsic NOTCH3 receptor expression. Modulation of NOTCH activity by immobilized ligand and by gamma‐secretase inhibitor demonstrates that Notch signaling is central in the pro‐myofibrogenic/antiadipogenic fate triggered by replicative senescence. The link between NOTCH3 and myofibrogenesis is further supported by gene expression studies on both human and mouse AT and analyses of human progenitor subsets.

## 2 METHODS

### 2.1 Human adipose tissues

Subcutaneous human AT was obtained from healthy adult women undergoing dermolipectomy (mean body mass index 27.5 kg/m^2^ ± SD 3.324, mean age 41.9 years ± SD 9.8). The protocol was approved by Ministère de la Recherche (DC2008‐452). Paired subcutaneous abdominal and visceral (omental) AT was obtained from patients, candidates for bariatric surgery through a clinical study approved by the Institutional Review Boards registered at ClinicalTrials.gov (SENAPID NCT01525472). All donors gave their informed consent.

### 2.2 Cell isolation and culture

AT was digested with dispase (2.4 U/ml in PBS, pH 7.4, volume/volume, Gibco) and then type I collagenase (250 U/ml in PBS, 2% BSA, pH 7.4, volume/volume, Sigma‐Aldrich) for 30 min at 37°C for the dermolipectomy, or with collagenase only for the SENADIP AT. Cell suspensions were filtered through a 250 μm filter. The stroma‐vascular cells (SVC) were obtained after centrifugation and treatment with erythrocyte lysis buffer (155 mmol/L NH_4_Cl; 5.7 mmol/L K_2_HPO_4_; 0.1 mmol/L EDTA; pH 7.3) for 10 min. Finally, matrix fragments were removed using successive filtrations through 100, 70, and 40 μm nylon meshes. The viable recovered cells were counted and further analyzed by flow cytometry or used to isolate the cell subsets by either magnetic beads‐based selection or cell sorter.

The CD45^−^/CD34^+^/CD31^−^ progenitor cells were obtained from SVC by elimination of the immune and endothelial cells using CD45^+^ depletion kit (Stemcell Technologies) followed by CD31^+^ depletion (R&D Systems or Dynal, Thermofisher), followed by CD34^+^ selection kit (Stemcell Technologies) according to the manufacturer's instructions. Purity of the progenitor cells was assessed by flow cytometry.

Cells were cultured at 37°C under 21% O_2_ and 5% CO_2_. For adipogenic differentiation, progenitor cells were seeded at high density (120,000 cells/cm^2^) in ECGM‐MV (Promocell) with 50 mg/ml penicillin–streptomycin for 48 h, trypsinated, and seeded in culture plates containing basal defined adipogenic medium (ECBM (Promocell) with 66 nmol/L insulin, 1 nmol/L triiodothyronine, 0.1 μg/ml transferrin, 100 nmol/L cortisol) supplemented with 3 μmol/L rosiglitazone and isobuthylmethylxantine (IBMX, 0.25 mmol/L) and precoated with human recombinant NOTCH ligands: IgG1‐Fc, JAG1‐Fc and DLL4‐F (Invitrogen) diluted in PBS and coated at 2 μg/cm^2^ overnight at 4°C or 3 h at room temperature (RT). After 3 days, the medium was replaced by basal defined adipogenic medium for the following 7 days. At day 10, cells were either fixed in 4% PFA solution at RT for 10 min or lysed for RNA extraction. For myofibrogenic differentiation, progenitor cells were seeded at high density (120,000 cells/cm^2^) in ECGM‐MV for 48 h, trypsinated, and seeded on coated recombinant NOTCH ligands in ECBM 1% fetal calf serum (FCS), supplemented or not with recombinant TGFβ1 (5 ng/ml, Peprotech), and treated or not with γ‐secretase inhibitor LY411575 (10 μmol/L, Sigma‐Aldrich) or TGFβ1 receptor inhibitor SB431542 (10 μmol/L, Sigma‐Aldrich) for 4 days. At day 4, cells were either fixed with 4% PFA or lysed for RNA extraction. For serial passaging, progenitor cells were seeded at low density (5000 cells/cm^2^) in ECGM‐MV, and then at 80% confluency, cells were trypsinated and seeded at low density for next passage, or at high density on coated ligand in ECBM 1% FCS. Immuno‐selected P1 progenitor cells were treated with hydrogen peroxide (H_2_O_2_) 0.1 μmol/L during 24 h, then lysed for RNA extraction or by doxorubicin (0.2 μM, Sigma‐Aldrich) or etoposide (1 μM, Sigma‐Aldrich) for 72 h, then either lysed for RNA extraction, or fixed with 4% PFA. For senolytic treatment, P3 and P6 progenitor cells were seeded at high density on IGG or DLL4 in ECGM‐MV and treated with dasatinib (50 nM, Sigma‐Aldrich) and quercetin (20 μM, Sigma‐Aldrich) for 4 days then lysed for RNA extraction. For conditioned media analyses, progenitor cells (native or passaged) were seeded at high density (120,000 cells/cm^2^) in ECGM‐MV for 24 h, followed by three washes with ECBM and incubated for 24 h within ECBM. Media were then collected and frozen at −80°C before mass spectrometry analyses. The progenitor cell subsets, that is, MSCA1^+^, −/CD271^+^ (MSCA1^−^/CD271^+^), and −/− (MSCA1^−^/CD271^−^) were isolated using a cell‐sorting approach as described previously (Esteve et al., 2019) and lysed for RNA extraction or cultured under myofibrogenic condition.

### 2.3 Senescence‐associated β‐galactosidase colorimetric staining

After fixation with 0.5% glutaraldehyde (5 min for cells and 10 min for tissue), tissue pieces or cells were incubated for the next 16 h with 4 mM K3Fe(CN)_6_, 4 mM K4Fe(CN)_6_, 2 mM MgCl2, and 400 μg/ml X‐Gal in C_3_H_7_NO (dimethylformamide) in PBS/MgCl2 pH 6.0 and after washes, examined by microscopy (microscope Nikon Eclipse TE300).

### 2.4 Senescence‐associated β‐galactosidase fluorescence staining and flow cytometry approaches

100,000 SVC were incubated with fluorescent‐labeled antibodies (V500‐CD45, PerCP‐CD34, and V450‐CD31 (BD Biosciences), PE‐MSCA1, APC‐CD271, and PE‐Vio770‐CD14 (Miltenyi Biotec)) or appropriate isotype control for 30 min at 4°C in PBS 0.5% BSA and 2 mmol/L EDTA. Cells were washed with PBS and analyzed using a FACS Canto™ II flow cytometer. Beta‐galactosidase activity was performed using ImaGene Green C_12_FDG lacZ Gene Expression Kit (Molecular Probes). 250,000 cells in ECBM 0.1% BSA without phenol red, pretreated with chloroquine (30 μmol/L) for 1 h at 37°C under 5% CO_2_, were further incubated for the next 3 h with 33 μmol/L FITC C_12_FDG. After washing steps, antibodies or appropriate isotype control were added (PerCP‐CD34, PE‐Vio770‐CD14, V450‐CD31, and BV510‐CD45) for 20 min at 4°C. Cells were washed with PBS and analyzed using a FACS Canto™ II flow cytometer. Analyses were performed with Diva Pro software (BD Biosciences) or FlowJo (BD Biosciences).

### 2.5 Scratch wound assay

p96 plate (Incucyte® Imagelock 96‐well plate, Sartorius, France) was coated overnight at 4°C with IgG and DLL4. Immuno‐selected progenitor cells were plated in ECGM‐MV at 30–40,000 cells/well on IgG/DLL4‐coated wells in the presence or not of 10 μmol/L of LY411575 or of 10 μmol/L of SB431542 or on uncoated wells, and left overnight. Scratch wound was performed according to the manufacturer's instructions using the 96‐well WoundMaker (Incucyte, Sartorius). After PBS washes, media were changed to ECBM 1% FCS in the presence or not of LY411575 (10 μmol/L), or in the presence of TGFβ1 (5 ng/ml), or SB431542 (10 μmol/L) and recorded every 3 h using the Incucyte SX5 during 72 h. Wound closure was analyzed using the Incucyte Scratch Wound Analysis Software Module (Sartorius, France).

### 2.6 Isolation of SVC from mouse AT

Four‐month‐old male C3H/HeOuJ mice (Charles River Laboratories France) were fed a 45% high‐fat diet (45% energy as fat, Research Diets D12451) up to 6 weeks. Mice were housed in accordance with French and European Animal Care Facility guidelines at 21 °C with food and water provided ad libitum, and maintained on a 12‐h light, 12‐h dark cycle. Mice were euthanized after an overnight fasting. Perigonadal AT was excised and directly digested as described previously (Vila et al., 2014). SVC were lysed in QIAzol (Qiagen) for RNA extraction.

### 2.7 Immunofluorescence staining

Fixed cells were blocked and permeabilized in PBS 0.1% Triton X‐100 and 5% serum for 30 min at RT and then incubated overnight at 4°C with primary antibodies (mouse anti‐αSMA, Dako 1A4, 1:100; rabbit anti‐NOTCH3, Abcam ab23426, 1:200, mouse anti‐γH2AX, EMD Millipore JBW301, 1:200). After washing steps, cells were incubated for 1 h at RT with appropriate Alexa Fluor‐conjugated secondary antibodies (Invitrogen). Nuclei were stained with DAPI (0.5 μg/ml, Invitrogen), and lipid accumulation was visualized with BODIPY 493/503 staining (10 μg/ml, Invitrogen) for 15 min at RT. Images were taken with a fluorescent (Nikon Eclipse TE300, software NIS‐Elements 2.5 BR, Nikon®) or confocal microscope (ZEISS LSM780, ZEN software). Image analysis was performed with ImageJ software. Nuclei were counted from the DAPI staining, and the results were the mean of 5 images for each sample.

### 2.8 Western blot analysis

Cells were lysed with RIPA buffer supplemented with antiproteases, and 2.5 μg of protein extract quantified by DC protein assay (Bio‐Rad) was used for the western approach. Primary antibodies rabbit anti‐NOTCH3 (Abcam ab23426, 1:1000), mouse anti‐β‐tubulin (Cell Signaling D3U1W, 1:1000, as loading control) were incubated overnight at 4°C in TBS buffer with 5% BSA or 5% milk. Detection was performed with an appropriate HRP‐coupled secondary antibody (Cell Signaling Technology) incubated for 1 h at RT and ECL reagent (Amersham). Densitometry was quantified using Chemidoc detection system (Bio‐Rad laboratories).

### 2.9 Transcriptional analysis

Total RNA was isolated from human cells using Quick‐RNA Microprep kit (Zymo Research) or from murine SVC using Direct‐zol RNA Miniprep Plus kit (Zymo Research) according to the manufacturer's instructions. cDNA synthesis was performed on 100–200 ng of total RNAs with Superscript III (Thermofisher) and random hexamer primers. Relative gene expression levels were assessed using Taqman® Probes and TaqMan Fast Advanced Applied mastermix (Thermofisher) on a QuantStudio™ 5 (QS5; Thermofisher). Human and murine Assay‐On‐Demand are listed in Table S1. Each of the samples was run in duplicate, and the relative amount was normalized to *18 S* or *PPIB* housekeeping gene. Data were analyzed using QuantStudio Real‐Time PCR software (Thermofisher).

### 2.10 Label‐free quantitative mass spectrometry

Adsorption of proteins on the surface of Nanozeolite LTL (NanoScape AG, Germany) was carried out for 90 min at 4°C by incubation of 0.1 mg protein/ml conditioned media and 0.1 mg/ml nanoparticles in 50 mmol/L ammonium bicarbonate buffer, pH 8.0 (ABF). After centrifugation at 16,000 g for 20 min and washes in ABF, captured proteins were suspended in 100 μl of ABF containing 0.05% AALS (Anionic Acid Labile Surfactants from Protea Biosciences). After reduction (10 mM DTT at 56°C for 30 min) and alkylation (20 mmol/L iodoacetamide for 30 min at RT in dark), bound proteins were digested with LysC (0.5 μg) for 4 h at 37°c followed by trypsin (1 μg) for 18 h at 37°C. After centrifugation, protein digests are collected and AALS hydrolyzed with 1% TFA at 37°C for 45 min. For LC–MSMS analysis of the native Sc and Vs progenitor cell secretome, Ultimate 3500 RSLC dual system (Thermo Scientific) coupled to hybrid LTQ Orbitrap Elite mass spectrometer (Thermo Scientific) equipped with a nanoelectrospray source was used. Tryptic digests were loaded onto a C18 trap column (Thermo Scientific) and washed with 0.2% HCOOH at 5 μl/min for 10 min. Peptides were eluted on a C18 reverse‐phase column (Thermo Scientific) with a linear gradient of 4%–30% solvent B (H_2_O/CH_3_CN/HCOOH, 10/90/0.2 volumes) for 120 min, 30%–90% solvent B for 20 min, and 90% solvent B for 5 min, at a flow rate of 250 nl/min. The mass spectrometer was operated in the data‐dependent mode to automatically switch between MS and MS/MS acquisition. Survey full‐scan MS spectra (m/z 310–1600) were acquired in the Orbitrap with a resolution of 120,000 at m/z 400. For serial passaging secretome, the nanoAcquity UPLC (Waters) coupled to a Q Exactive Plus mass spectrometer (Thermo Scientific) equipped with a nanoelectrospray source was used. Protein digests were loaded onto a nanoAcquity UPLC Trap column (Waters) and washed with 0.2% formic acid at 20 μl/min for 3 min. Peptides were then eluted on a C_18_ reverse‐phase nanoAcquity column (Waters) with a linear gradient of 8–31% solvent B (H_2_O/CH_3_CN/HCOOH, 10/90/0.2, by vol.) for 120 min, 31–91% solvent B for 20 min, and 91% solvent B for 5 min, at a flow rate of 250 nl/min. The mass spectrometer was operated in the data‐dependent mode to automatically switch between MS and MS/MS acquisition. Survey full‐scan MS spectra (from m/z 325–1300) were acquired with a resolution of 70,000 at m/z 200. MSMS spectra were recorded in profile type with a resolution of 17′500. All samples were injected in triplicate. The LC–MS/MS data, acquired using the Xcalibur software (Thermo Fisher Scientific), were processed using a homemade Visual Basic program software developed using XRawfile libraries (Thermo Fisher Scientific) to generate an MS/MS peak list used for database searching and a file used for quantitative analysis. Database searches were performed using internal MASCOT server (Matrix Science) using the Swiss‐Prot human database. Mascot results were imported into Scaffold software (version 4.4.1.1) and also used for XTandem parallel Database Search. Peptide identifications were accepted if they could be established at greater than 7.0% probability to achieve an FDR less than 1.0% by the Scaffold Local FDR algorithm. Protein identifications were accepted if they could be established at greater than 97.0% probability to achieve an FDR less than 1.0% and contained at least 2 identified peptides. Quantitative differential analysis of proteins was realized using a label‐free analysis with an in‐house DIFFTAL (DIFferential Fourier Transform Analysis) software algorithm as described previously (Autelitano et al., 2014). Statistical analyses were realized with DanteR program, an R‐based software. The peptides quantification was normalized by the median intensity value of the detected feature population over the 102 runs, which includes 34 conditions (17 patients × 2 AT depots) and over the 36 runs, which includes 12 conditions (3 patients × 4 levels of serial passaging), in triplicate injections. Only peptides detected at least 2 times (over replicates) are kept, and an average intensity value per sample is calculated for each peptide. A threshold value representing the minimum detectable signal level is used. The protein abundance is determined as the average of the top three most abundant peptides per protein.

### 2.11 Ampliseq RNA sequencing

RNA was isolated using RNeasy Protect Mini kit (Qiagen) and suspended in RNAprotect cell reagent. RNA quality was assessed with Agilent RNA 6000 Pico Kit (Agilent) and quantified using Qubit™ RNA HS Assay Kit (Invitrogen) and Qubit 2 Fluorometer (Invitrogen). 10 ng of RNA was used for AmpliSeq transcriptome library construction. For AmpliSeq transcriptome sequencing library construction AmpliSeq™ Library PLUS, AmpliSeq Transcriptome Human Gene Expression Panel and AmpliSeq CD indexes SetA kits were purchased from Illumina and sequencing libraries were constructed as described in AmpliSeq for Illumina Transcriptome Human Gene Expression Panel reference guide (Illumina). Sequencing libraries were quantified using Qubit dsDNA HS Assay Kit and Fluorometer (Invitrogen) and converted to molar concentration using 285‐bp peak. Equimolar concentrations of libraries were pooled at 4 nmol/L and used for sequencing. Pooled library was denatured and diluted as described in Denature and Dilute Libraries Guide (Illumina) and adjusted to final concentration of 1.4 pmol/L. Resulting library was sequenced on NextSeq 500 using NextSeq 500/550 High Output v2 kit with 2 × 151 bp cycle. Generated raw files were converted to FASTQ files and used for data analysis. AmpliSeq transcriptome FASTQ files were analyzed on Array studio V10.0 (Omicsoft, Qiagen). Following raw read QC, first and last 10 bases were trimmed and mapped to reference genome Human B38. The read count data were generated using GeneModel RefGene20170606. Resulting data were normalized by DESeq package, transformed to log2 value, and used for statistical analysis using NetworkAnalyst (Zhou et al., 2019).

### 2.12 Statistical analyses

Statistical analyses were performed using Prism (GraphPad Software). Comparisons between 2 groups were analyzed either paired or unpaired analysis and two‐tailed Student's *t* test or Wilcoxon test depending on data distribution. Comparison between more than 2 groups was analyzed by either one‐way ANOVA or two‐way ANOVA or nonparametric Kruskal–Wallis test, followed by Dunn's, Dunnet's, Tukey's, or Sidak's multiple comparison test for (n) independent experiments. Differences were considered statistically significant when *p* < 0.05.

## 3 RESULTS

### 3.1 Fat depot location from obese patients impacts senescence of progenitors

**FIGURE 1:**
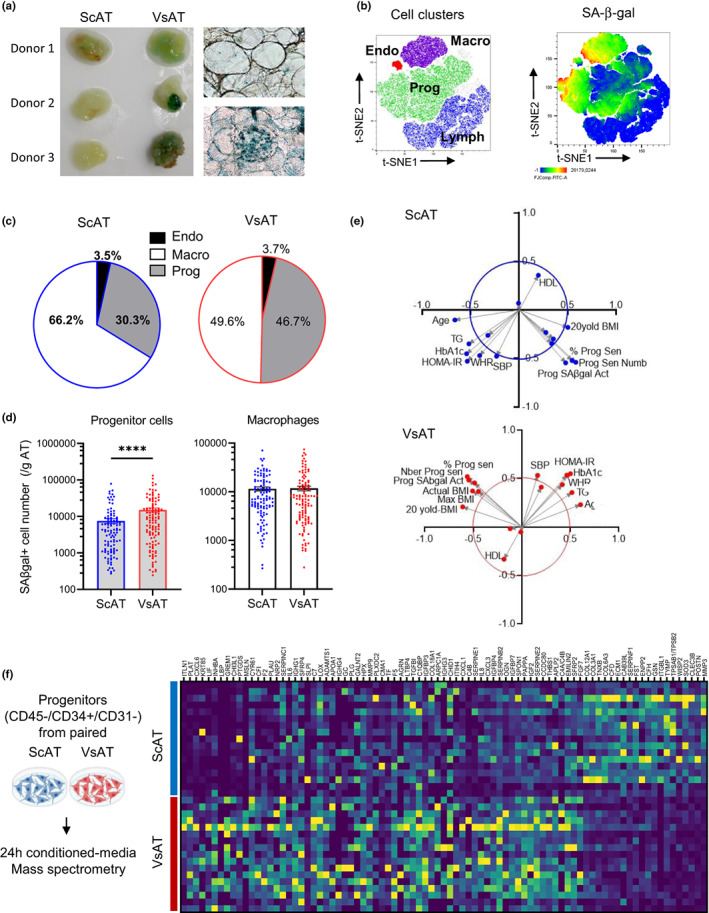
Senescence in progenitor cells defines a fat depot‐specific pattern in obesity. (a) Colorimetric SA‐β‐gal staining on paired biopsies of subcutaneous and visceral adipose tissue (ScAT and VsAT). Representative views of whole AT pieces and light microscope images are shown. (b) Paired ScAT and VsAT stroma‐vascular fraction (SVF) cells from 55 subjects were analyzed by flow cytometry. tSNE of data after downsampling and concatenation enabled the identification of clusters by cell type: purple macrophages (CD45^+^/CD34^−^/CD31int/highFSC/HighSSC), red endothelial cells (CD45^−^/CD34^+^/CD31^+^/HighFSC/HighSSC), green progenitor cells (CD45^−^/CD34^+^/CD31^−^/HighFSC/HighSSC), and blue lymphocytes (CD45^+^/CD34^−^/CD31^−^/lowFSC/LowSSC; left panel) and same tSNE colored by SA‐β‐gal fluorescence intensity (right panel). (c) Pie charts showing the percentage of cell types within the SA‐β‐gal‐positive cells in ScAT and VsAT. (d) Supervised analysis of the SA‐β‐gal+ cells, histograms represent means ± sem of SA‐β‐gal+ cells per gram of tissue for progenitor cells (left) and macrophages (right) from ScAT (blue) and VsAT (red; *n* = 115 donors, two‐way ANOVA, Tukey's post‐test). (e) Principal component analyses performed on clinical and anthropometrical parameters of obese AT donors and ScAT (upper panel, blue) and VsAT (lower panel, red) progenitor senescence variables. (f) Immuno‐selected progenitor cells from paired ScAT and VsAT biopsies of 17 obese subjects were maintained for 24 h in basal medium, and the conditioned media were harvested and analyzed by mass spectrometry. Heatmap of most relevant differentially expressed proteins from the 17 secretomes of ScAT (blue) and VsAT (red) progenitors (deep blue corresponding to the smallest value and yellow to the highest value)

Cellular senescence in both ScAT and VsAT from obese patients was first assessed through colorimetric staining of senescence‐associated beta‐galactosidase (SA‐β‐gal) activity. As shown in Figure 1a, SA‐β‐gal staining showed interindividual and interdepot heterogeneity, exhibiting a range of distinct qualitative profiles, from a diffuse low‐level staining in ScAT biopsies to much higher staining in VsAT (left panel), with positive cells widely distributed in the stroma‐vascular compartment or concentrated in crown‐like structures surrounding adipocytes (right panel, top and bottom, respectively). Although some mature adipocytes exhibited light SA‐β‐Gal staining, more intense SA‐β‐Gal staining was found in stromal cells. To further identify and quantify SA‐β‐gal‐positive stromal cells in human AT, stroma‐vascular cells obtained after collagenase digestion of both ScAT and VsAT from 115 obese subjects were stained for fluorescent SA‐β‐gal activity in combination with cell surface markers (CD45, CD14, CD34, and CD31) and analyzed by flow cytometry. Unsupervised tSNE (t‐distributed stochastic neighbor embedding) performed after downsampling and concatenation of ScAT and VsAT data from a panel of 55 donors highlighted macrophage, lymphocyte, endothelial cell and progenitor cell clusters based on cell surface marker expression (Figure S1a,b left panel). SA‐β‐gal+ cells were identified in macrophages, endothelial cells, and progenitor cells (Figure 1b right panel). Within the total SA‐β‐gal+ stromal cells, VsAT had higher percentage of progenitors and lower of macrophages than ScAT (Figure 1c). Per tissue mass, VsAT contained a higher number of SA‐β‐gal+ senescent progenitors when compared to ScAT, with no corresponding difference in the number of senescent macrophages (Figure 1D). In the progenitor cluster, back‐gating on the fat depot origin revealed higher SA‐β‐gal activity together with larger cell size in VsAT compared with ScAT (Figure S1b–d). Principal component analysis using clinical and anthropometric variables in addition to ScAT and VsAT progenitor senescence variables was performed (Figure 1e). Component 1 clearly separated two groups of variables. One group was constituted by the variables relative to progenitor senescence and BMI (at the age of 20, actual and maximal BMI). The other group was composed of parameters related to metabolic syndrome (MS) including age, waist‐to‐hip ratio (WHR), insulin resistance (HbA1c and HOMA‐IR), triglycerides, and systolic blood pressure (SBP). HDL was clearly discriminated by component 2 and, as expected, was inversely correlated with the MS‐related variables. Interestingly, the associations between BMI and senescent parameters were clearly more marked and statistically relevant in VsAT compared with ScAT (Figure 1e).

Analysis of the secretome of native immuno‐selected ScAT and VsAT progenitor cells from 17 patients by large‐scale mass spectrometry identified 428 secreted proteins. Paired analysis of ScAT and VsAT progenitor secretomes highlighted 161 differentially expressed proteins (DEPs) upregulated in VsAT progenitors and 65 in ScAT progenitors; the DEPs with the highest fold changes are shown in Figure 1f. Comparison of ScAT and VsAT DEPs with the freely available secretome database, the SASP atlas (Basisty et al., 2020), the SenMayo geneset (Saul et al., 2022) and factors identified in human AT senescent progenitors (Xu, Palmer, et al., 2015; Xu, Tchkonia, et al., 2015) were performed. VsAT progenitor secretome was mainly enriched in common canonical SASP factors including IGFBP7, IL6, and CXCL8 (cellular senescence‐enriched reactome pathway), the CXCR2 Ligands (CXCL1, CXCL2, CXCL3, and CXCL8), as well as inflammatory, growth, and remodeling factors (MIF, FGF7, and INHBA) and factors of the complement and coagulation pathways (PLAT, PLAU, SERPINB2, E2 and E1, and TF; Table 1).

**TABLE 1:** Differentially expressed proteins in ScAT and VsAT secretomes, SASP atlas, SenMayo gene set and media from irradiated human preadipocytes

Gene Id	*p* Value	Ratio	SASP Atlas				SenMayo	Irradiated preadipocytes
			Irradiated fibroblasts	Protease inhibitor fibroblasts	Inducible RAS Fibroblast	Irradiated epithelial cells		
ACE	0.001953	0.65						
ADAMTS1	0.000427	57.57						
ADAMTS5	0.03125	0.82						
AEBP1	0.044769	0.88						
AGRN	0.03125	29.43	+	+	+			
ALB	0.003845	1.96	+		+			
ANXA2	0.001678	0.81		+	+	+		
APCS	0.012207	1.75						
APLP2	0.005569	2.25						
APOA1	0.00293	55.76			+			
APOC3	0.009766	1.60			+			
APOD	0.000153	0.52						
ARPC1A	0.03418	22.82						
B4GALT1	0.028992	1.39		+				
BGN	0.003159	1.76		+	+			
C1QBP	0.000381	25.15						
C4A	0.007813	2.19						
C4B	0.003906	4.58						
C7	0.000031	65.24						
CAB39L	0.000488	0.44						
CALR	0.000015	1.96	+	+	+			
CCDC80	0.00029	2.35						
CD59	0.006653	1.28			+			
CFD	0.000107	0.44						
CFH	0.000031	0.39		+				
CFI	0.000488	230.90			+			
CHI3L1	0.000122	294.27						
CHID1	0.002441	20.90						
CILP	0.002441	0.60						
CLEC3B	0.000031	0.25		+	+			
CMA1	0.04187	37.97						
COL12A1	0.000031	0.50	+	+	+			
COL14A1	0.003906	0.60						
COL18A1	0.010742	23.76		+				
COL1A1	0.000381	0.65		+				
COL1A2	0.003845	0.71		+				
COL3A1	0.000107	0.48		+				
COL5A2	0.028992	0.91			+			
COL6A1	0.00029	0.61	+	+	+			
COL6A2	0.000153	0.59	+	+	+			
COL6A3	0.000153	0.45	+	+	+			
CPA3	0.010986	0.80						
CREG1	0.00029	1.92						
CTHRC1	0.006836	0.67	+		+			
CXCL1	0.003845	6.44	+	+	+		+	+
CXCL2	0.027344	1.83	+	+	+		+	
CXCL3	0.008545	3.07	+	+	+			
CXCL6	0.000092	735.04					+	
CXCL8	0.000854	3.11	+		+		+	+
CYR61	0.038635	233.45		+	+			
DCN	0.001343	0.60		+		+		
ECM1	0.000031	0.44	+	+	+			
EMILIN2	0.000076	2.12						
ENPP2	0.000488	0.39		+				
F2	0.000061	195.33			+			
F5	0.000214	32.05		+				
FAM114A1	0.03125	0.85						
FBLN1	0.000381	0.50		+	+			
FBLN2	0.000381	0.59		+				
FBN1	0.000504	0.59		+	+			
FGF7	0.001953	2.01					+	
FGG	0.03125	1.44						
FN1	0.020157	0.79	+	+	+			
FST	0.000092	0.41						
FSTL1	0.001343	0.67		+				
GALNT2	0.037109	48.98	+					
GC	0.003906	53.89	+		+			
GGH	0.020996	0.87						
GNAS	0.005157	1.29						
GREM1	0.000977	324.39						
GSN	0.000015	0.37	+	+	+	+		
HMCN1	0.013672	0.75						
HP	0.010986	1.83						
HPX	0.001678	48.49						
IGF2	0.000061	2.42		+	+			
IGFBP3	0.000015	24.82	+	+		+	+	
IGFBP4	0.000504	2.79	+	+	+		+	
IGFBP7	0.000031	2.54	+	+			+	
IGHG1	0.000214	85.05						
IGHG3	0.023438	22.28						
IGHG4	0.015625	54.44						
IGKC	0.006653	1.96						
IL6	0.000732	93.41					+	+
INHBA	0.000031	338.34					+	+
ISLR	0.023438	0.83						
ITGBL1	0.000977	0.37						
ITIH4	0.020996	19.22						
ITLN1	0.000244	1087.89						
KRT85	0.027344	510.83						
LAMB1	0.000656	1.65	+	+	+			
LAMB2	0.000381	0.63		+	+	+		
LBP	0.03125	330.45						
LGALS1	0.000656	0.74	+	+	+			
LGALS3	0.000015	0.54	+	+		+		
LGALS3BP	0.039536	1.47	+	+	+			
LIF	0.000122	402.14						
LOX	0.003357	58.52		+				
LOXL2	0.023438	0.84	+	+	+			
LTBP2	0.001007	1.78			+			
LTBP4	0.047913	28.77			+			
MASP1	0.007813	0.58		+				
METRNL	0.024414	0.87						
MFAP5	0.015625	0.69						
MIF	0.000046	1.69	+	+	+		+	
MMP10	0.001678	0.56					+	
MMP14	0.029541	1.98			+		+	
MMP3	0.000015	0.18			+		+	+
MMP9	0.003159	45.56			+		+	
MSLN	0.03125	249.28						
MYDGF	0.003357	1.48	+		+			
NAMPT	0.014999	0.81	+		+			
NID2	0.005859	1.69	+		+			
NRP2	0.001953	147.98						
OGN	0.000122	2.59						
PAM	0.044312	0.90						
PAMR1	0.01825	1.75						
PAPPA	0.000031	2.51					+	
PCOLCE	0.007904	0.74		+	+			
PCOLCE2	0.000153	0.57						
PDGFRL	0.002441	0.69						
PI3	0.044769	0.97						
PLAT	0.000031	972.35			+		+	
PLAU	0.000031	176.63	+		+		+	
PLG	0.013672	51.15		+				
PLXDC2	0.027344	41.60						
POSTN	0.000015	0.24	+	+	+			
PRG4	0.00209	0.64						
PSAP	0.026672	1.32	+	+	+			
PTGDS	0.001068	268.45						
PXDN	0.047913	1.30	+	+	+			
QSOX1	0.001678	1.39	+	+	+			
RNASE4	0.024414	0.79		+	+			
S100A13	0.000031	0.52						
SDC4	0.001678	1.74						
SEPT8	0.039795	1.27						
SERPINB2	0.000107	2.75			+			
SERPINC1	0.001068	125.93						
SERPINE1	0.000015	3.30	+	+	+		+	
SERPINE2	0.000504	2.39		+			+	
SERPINF1	0.000031	0.43		+	+			
SERPING1	0.030518	0.82		+				
SFRP2	0.038635	2.02						
SFRP4	0.047913	83.82						
SLIT2	0.007813	0.72						
SLPI	0.010254	70.58						
SOD3	0.000015	0.25		+				
SPON1	0.01825	2.53				+		
STC2	0.006714	1.84				+		
TF	0.002136	34.82						
TGFBI	0.03479	28.33	+	+				
TGFBR3	0.026672	1.78						
THBS1	0.000153	2.35	+	+	+			
THBS2	0.000839	1.93		+	+			
TNXB	0.000076	0.48						
TPSAB1/TPSB2	0.000061	0.25						
TXN	0.001068	0.75			+			
TYMP	0.000015	0.26						
VCAN	0.000046	0.55	+	+	+			
VTN	0.000763	1.82						
WISP2	0.000061	0.25						
YBX1	0.030151	0.88	+		+			

*Note*: Proteins were selected from available datasets (SASP atlas) as statistically upregulated by senescence stimuli in conditioned media.

### 3.2 The BMP, WNT, and NOTCH developmental pathways are markers of the senescence‐associated secretory and intrinsic phenotypes of progenitors

**FIGURE 2:**
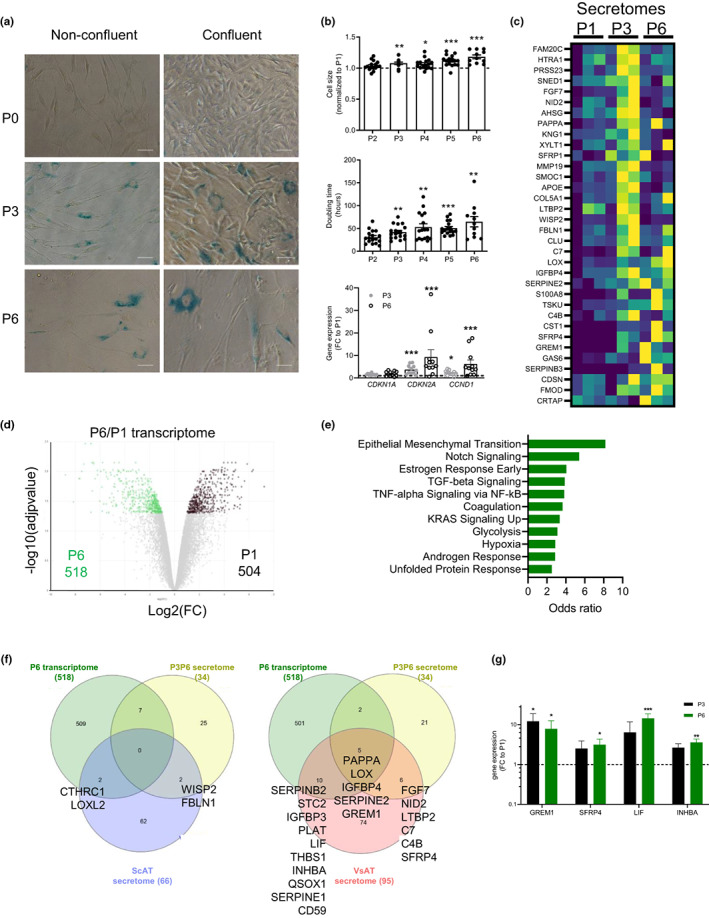
Replicative senescence‐associated secretory phenotype of AT progenitor cells highlights components of the TGFβ/BMP and WNT developmental pathways. (a) SA‐βgal activity by colorimetric staining on nonconfluent or confluent progenitors at Passage 0 (P0), 3 (P3), and 6 (P6). Representative photomicrographs from *n* = 3 donors are shown. Scale bars: 50 μm. (b) Changes in cell size according to increasing passages. Results are expressed as fold change over P1, means ± SEM of *n* = 11–17 independent experiments (one‐way ANOVA, Dunnett's post‐test, *p < 0.05, **p < 0.01, ***p < 0.001 compared with P1); changes in doubling time according to increasing passages. Values are means ± SEM of *n* = 11–17 independent experiments (one‐way ANOVA, Dunnett's post‐test, **p < 0.01, ***p < 0.001 compared with P2, changes in mRNA expression of *CDKN2A*, *CDKN1A*, and *CCND1* at P3 and P6. Results are expressed as fold change over P1, means ± SEM of *n* = 15 independent experiments (one‐way ANOVA, Dunn's post‐test, ***p < 0.001 compared with P1). (c) Heatmap of differentially expressed proteins in paired analysis between P1 and P3 and P1 and P6 secretomes from three donors, (deep blue corresponding to the smallest value and yellow to the highest value). (d) Volcano plot of the twofold and more differentially expressed genes between P1 and P6 RNA sequencing transcriptomes performed in three donors. (e) Statistically significantly enriched pathways in the 518 DEGs upregulated in P6 transcriptome in the Human Molecular Signatures Database (MSigDB) hallmark gene sets MSigDB Hallmark (GSEA). (f) Venn diagram of DEGs upregulated in the P6 transcriptome, P3 and P6P3 secretome and ScAT (left) and VsAT (right) native progenitor‐specific secretomes. (g) mRNA levels of SASP proteins *GREM1*, *SFRP4*, *LIF,* and *INHBA* determined in P1, P3, and P6 progenitor cells by RTqPCR. Results are expressed as fold change over P1, means ± SEM of *n* = 7 donors (*p < 0.05, **p < 0.01, ***p < 0.001 compared with P1)

To investigate whether the VsAT progenitor senescence could be in part related to replicative growth pressure promoted by obesity, AT progenitors from nonobese individuals were submitted to serial passaging with low‐density cell seeding. Due to the low amount of VsAT biopsies retrieved from nonobese individuals precluding immunoselection approaches, progenitors were immuno‐selected from ScAT. A marked increase in the number of SA‐β‐gal+ progenitor cells was observed as early as P3 in both nonconfluent and confluent cells (Figure 2a). In addition, increases in cell size and doubling time and in the expression levels of *CDKN2A* (p16) and *CCND1* (Cyclin D1) were observed while *CDKN1A* levels (p21) were not altered (Figure 2b). Paired analysis of the DEPs between P3 and P6 secretomes with the one of nonsenescent P1 progenitor cells identified 34 proteins upregulated either in P3 or P6 secretomes (Figure 2c) among the 342 identified secretory factors. In parallel, the comparative paired analysis of the RNA sequencing data of P6 and P1 progenitors highlighted 518 DEGs upregulated at P6 (Figure 2d). The P6 DEGs were enriched in pathways related to tissue remodeling including epithelial‐to‐mesenchymal transition (EMT), TGFβ signaling, and hypoxia as well as inflammation. Interestingly, the Notch signaling including NOTCH3 was also part of the AT progenitors senescent transcriptome (Figure 2e). Merging of the P6 DEGs and P3P6 DEPs with the native ScAT and VsAT progenitor‐specific secretomes from obese patients (from Figure 1f) highlighted a marked enrichment in senescence‐related proteins in VsAT compared with ScAT secretomes (21 proteins vs. 4, respectively; Figure 2f). Among the 21 VsAT SASP factors, 16 proteins were common with other SASP (Table 1). The remaining 5 proteins were specific of the AT progenitor replicative SASP and enriched in complement and coagulation pathways (C7 and C4B) as well as in TGFβ/BMP and Wnt/β‐catenin signaling, as GREM1 and SFRP4 are both secreted antagonists of BMP and WNT, respectively, and in adipogenesis‐related pathways (SFRP4 and LIF). RTqPCR analysis further confirmed the upregulation of *GREM1*, *SFRP4,* and *LIF* as well as *INHBA* with AT progenitor replicative senescence (Figure 1g).

### 3.3 The activity of Notch modulates the replicative SASP of human AT progenitors

**FIGURE 3:**
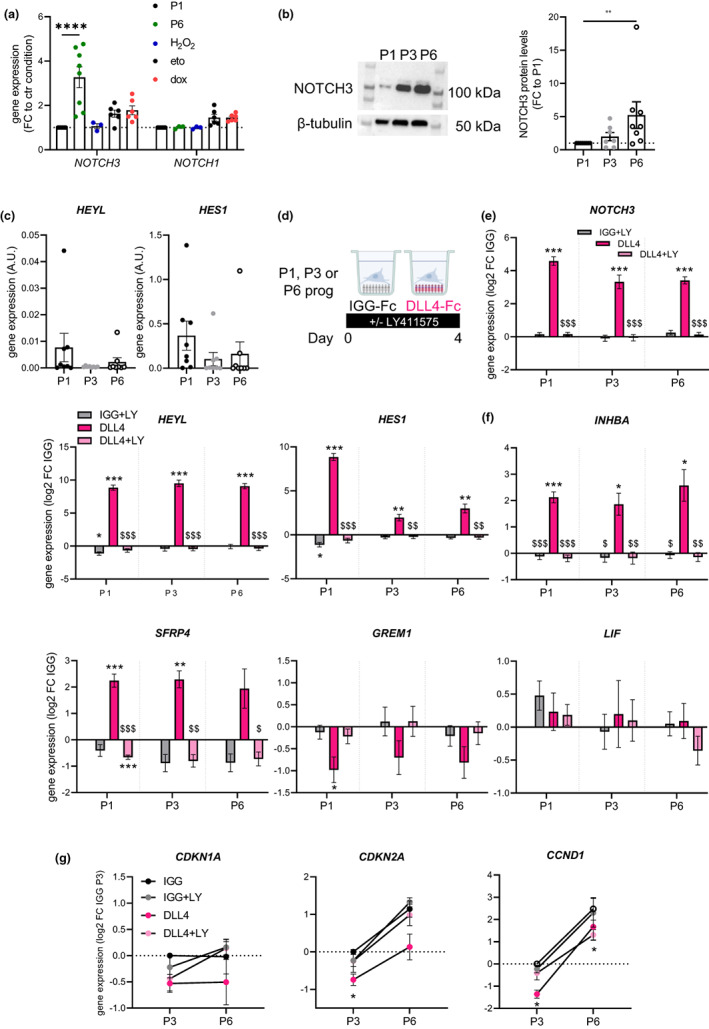
Replicative senescence upregulates the expression of Notch3 and does not alter the responses to Notch activation. (a) *NOTCH3* and *NOTCH1* transcript levels in progenitor cells at P1, at P6, after H_2_O_2_ or etoposide (eto) or doxorubicin (dox) treatments, determined by RTqPCR and RNAseq (H_2_O_2_ treatment) and normalized to respective P1 control cells (values are means ± SEM of *n* = 3–8 donors, two‐way ANOVA, Dunnett's post‐test, ****p < 0.0001). (b) NOTCH3 protein level in progenitor cells at P1, P3, and P6 determined by western blot and quantification of the ratio between the shorter and the higher cleaved form normalized to the β‐tubulin level. Results are expressed as fold change over P1, means ± SEM of *n* = 7–8 donors (one‐way ANOVA, Dunn's post‐test, **p < 0.01). (c) mRNA levels of *HEYL* and *HES1* in progenitor cells at P1, P3, and P6 determined by RTqPCR (values are means ± SEM of *n* = 8 distinct donors, one‐way ANOVA, Dunn's post‐test). (d) Immuno‐selected ScAT P1, P3, or P6 progenitor cells were seeded on immobilized IGG control or NOTCH ligand DLL4 and cultured in basal medium supplemented or not with gamma‐secretase inhibitor LY411575 (LY) for 4 days. mRNA levels of (e) *NOTCH3* and NOTCH target genes *HEYL* and *HES1*, (f) SASP factors *INHBA*, *SFRP4*, *GREM1,* and *LIF* determined at day 4 by RTqPCR. Results are expressed as log2 fold change over IGG, means ± SEM of *n* = 7 donors (two‐way ANOVA, Tukey's post‐test, *p < 0.05, **p < 0.01, ***p < 0.001 compared with IGG, $*p* < 0.05, $$*p* < 0.01, $$$*p* < 0.001 compared with DLL4). (g) mRNA levels of senescence markers *CDKN1A*, *CDKN2A,* and *CCND1* in P3 and P6 progenitor cells determined at day 4 by RTqPCR. Results are expressed as log2 fold change over IGG P3, means ± sem of *n* = 3–4 donors (two‐way ANOVA, Dunnett's post‐test)

To further investigate the Notch‐dependent pathway highlighted in the replicative senescent AT progenitor transcriptome, ScAT progenitors were submitted to additional senescence‐promoting stimuli, including oxidative stress induced by H_2_O_2_ treatment and DNA damage induced by doxorubicin or etoposide treatments. The marked upregulation of *NOTCH3* transcript levels, detected by RTqPCR (confirming the RNA sequencing data), was only observed in P6 progenitors although both doxorubicin and etoposide increased the expression of senescent markers including *CDKN1A* and *CCND1* and γH2AX nuclear foci (Figure 3a and Figure S2). Interestingly, the effect of replicative senescence was specific for *NOTCH3,* as *NOTCH1* remained unchanged (Figure 3a). The increased level of *NOTCH3* mRNA with replicative senescence was associated with an increased level of the cleaved form of NOTCH3 protein with the apparent molecular weight of 100 kDa (Figure 3b). The basal levels of the canonical Notch target genes *HEYL* and *HES1* were not changed with replicative senescence (Figure 3c). To investigate the potential involvement of Notch pathway in the modulation of the replicative SASP, pharmacological approaches were performed to activate or inhibit Notch signaling in nonsenescent and senescent progenitors. Activation of Notch signaling was achieved by immobilized Notch ligand DLL4 (a member of the Delta‐like family) while inhibition by treatment with the γ‐secretase inhibitor LY411575, which prevents Notch cleavage (Figure 3d). As expected, Notch activation efficiently increased the mRNA level of Notch target genes *HEYL* and *HES1* as well as *NOTCH3* itself at P1. The stimulatory effects were completely inhibited by the presence of LY411575. Similar effects were observed in P3 and P6 progenitors, clearly showing that the senescent state did not alter Notch responsiveness (Figure 3e). Interestingly, Notch activation by DLL4 did not affect the expression of *GREM1* nor the one of *LIF*. *SFRP4* and *INHBA*, on the contrary, were upregulated regardless of the cellular state of senescence and in a γ‐secretase‐dependent manner (Figure 3f), indicating that Notch acted upstream of the SASP factors SFRP4 and INHBA. Such an effect was not related to a pro‐senescent impact of Notch activation since it did not increase but even rather slightly decreased the expression of both *CDKN2A* and *CCND1* at P3 and CCND1 at P6 without affecting the expression level of *CDKN1A* (Figure 3g).

### 3.4 NOTCH activation shifts progenitor cell fate balance toward myofibrogenesis over adipogenesis

To investigate the impact of Notch signaling on progenitor differentiation, immuno‐selected progenitor cells (P1) were seeded on immobilized NOTCH ligands JAG1 (a member of the Jagged family) or DLL4 and maintained under adipogenic or myofibrogenic culture conditions.

**FIGURE 4:**
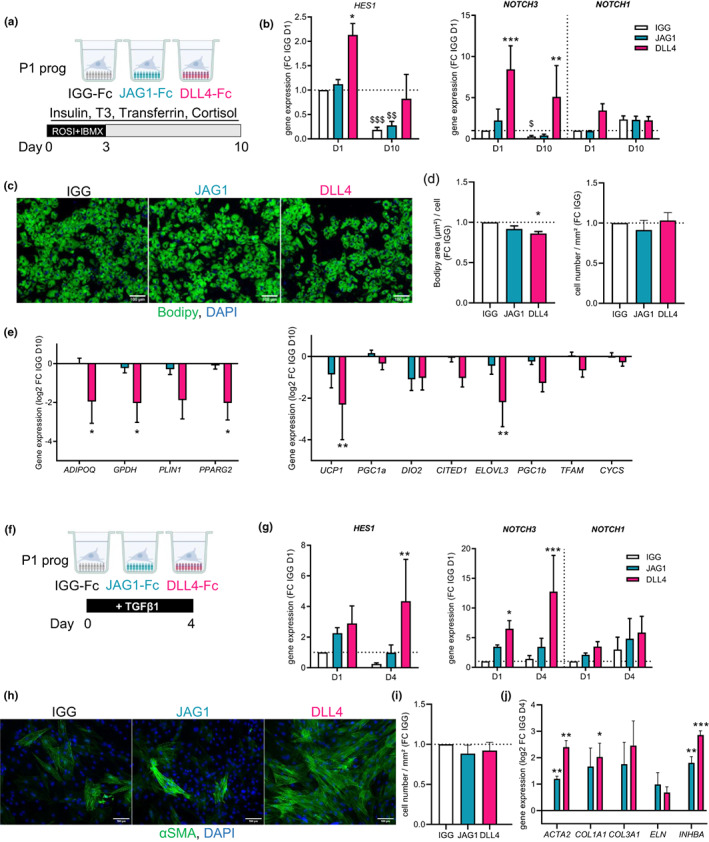
NOTCH activation inhibits adipogenesis and promotes myofibrogenesis. (a) Immuno‐selected ScAT P1 progenitor cells were seeded on immobilized IGG control (white) or NOTCH ligands JAG1 (cyan) or DLL4 (pink) and cultured in adipogenic medium for 10 days. (b) mRNA levels of *HES1*, *NOTCH3,* and *NOTCH1* determined at day 1 and day 10 by RTqPCR. Results are expressed as fold change over day 1 IGG control, means ± SEM of *n* = 6 donors (two‐way ANOVA, Dunnett's, and Sidak's post‐test, $$*p* < 0.01, $$$*p* < 0.001 compared with respective treatment at day 1). (c) Representative photomicrographs of Bodipy (green) and DAPI (blue) stainings at day 10 (scale bar = 100 μm). (d) Quantification of Bodipy area per cell (left panel) and cell number (right panel) at day 10, results are expressed as fold change over day 10 IGG control, means ± SEM of *n* = 3 donors (one‐way ANOVA, Dunnett's post‐test). (e) mRNA levels of adipocyte markers (*ADIPOQ*, *GPDH*, *PLIN1*, and *PPARG2*) and brite adipocyte markers (*UCP1*, *PGC1A*, *DIO2*, *CITED1*, *ELOVL3*, *PGC1B*, *TFAM*, and *CYCS*) determined at day 10 by RTqPCR. Results are expressed as log2 fold change over IGG control, means ± SEM of *n* = 6 donors (two‐way ANOVA, Dunnett's post‐test). (f) Immuno‐selected ScAT P1 progenitor cells were seeded on immobilized IGG control or NOTCH ligands JAG1 or DLL4 (colored as in a) and cultured in the presence of TGFβ1 during 4 days. (g) mRNA levels of *HES1*, *NOTCH3,* and *NOTCH1* determined at day 1 and day 4 by RTqPCR. Results are expressed as fold change over day 1 IGG control, means ± SEM of *n* = 4 donors (two‐way ANOVA, Tukey's post‐test). (h) Representative photomicrographs of αSMA (green) and DAPI (blue) staining at day 4 (scale bar = 50 μm). (i) Quantification of cell number at day 4. Results are expressed as fold change over IGG, means ± SEM of *n* = 5 independent experiments (one‐way ANOVA, Dunnett's post‐test). (j) mRNA levels of myofibroblast markers (*ACTA2*, *COL1A1*, *COL3A1*, *ELN*, and *INHBA*) determined at day 4 by RTqPCR. Results are expressed as log2 fold change over IGG, means ± sem of *n* = 4 donors (two‐way ANOVA, Dunnett's post‐test). *p < 0.05, **p < 0.01, ***p < 0.001 compared with IGG

Under adipogenic conditions (Figure 4a), DLL4 and JAG1 ligands had different effects on NOTCH3 activation. Stimulation with DLL4, and not with JAG1, led to a rapid and transient increase in the Notch target gene *HES1* at day 1. By contrast, DLL4 led to a marked induction of *NOTCH3* mRNA at day 1, which remained elevated until day 10, while the expression of *NOTCH1* was weakly increased only at day 1 (Figure 4b). Notch activation by DLL4 led to a mild, albeit significant, decrease in adipogenesis, as shown by the decrease in lipid‐laden cells (stained with Bodipy), without changing the number of cells (Figure 4c,d). It also reduced the expression of adipogenic genes (*ADIPOQ*, *GPDH*, *and PPARG2*) and brite adipogenic genes (*UCP1* and *ELOVL3*; Figure 4e). Adipogenic differentiation per se decreased the expression of *NOTCH3* and tended to increase the one of *NOTCH1* (Figure 4b). Likewise, isolated mature adipocytes expressed lower levels of *NOTCH3* and higher levels of *NOTCH1* compared with progenitor cells (Figure S3). Under myofibrogenic culture conditions in the presence of TGFβ (Figure 4f), DLL4 but not JAG1 induced an increase of *HES1* and *NOTCH3* mRNA levels, which remained elevated until day 4 (Figure 4g). *NOTCH1* expression was not significantly modulated under the same conditions (Figure 4g). Notch activation by DLL4 further enhanced myofibrogenesis, as shown by the increase in αSMA‐positive cells and the expression of myofibroblast genes (*ACTA2*, *COL1A1*, and *INHBA*; Figure 4h,j) with no impact on total cell number (Figure 4I). Analyses of RNAseq data retrieved from whole ScAT and VsAT from the GTEx database (https://www.gtexportal.org) showed that *NOTCH3* transcript levels were positively correlated with the ones of myofibrogenic‐ and SASP‐related genes such as *ACTA2*, *COL1A1*, *COL3A1*, *INHBA*, *GREM1,* and *SFRP4* and with TGFβ and EMT‐related transcription factor *SNAI2* but not *SNAI1*. Conversely, *NOTCH3* levels were negatively correlated with the expression of the adipogenic‐related markers *ADIPOQ*, *GPDD1*, *PLIN1,* and *PPARG*. Similar trends were observed for both ScAT and VsAT, but correlations were weaker in VsAT. These observations were specific for *NOTCH3* as the correlation with *NOTCH1* transcript levels exhibited the opposite pattern (Figure S4a,b).

### 3.5 NOTCH activation induces myofibrogenesis without exogenous addition of TGFβ and remains active in senescent cells

**FIGURE 5:**
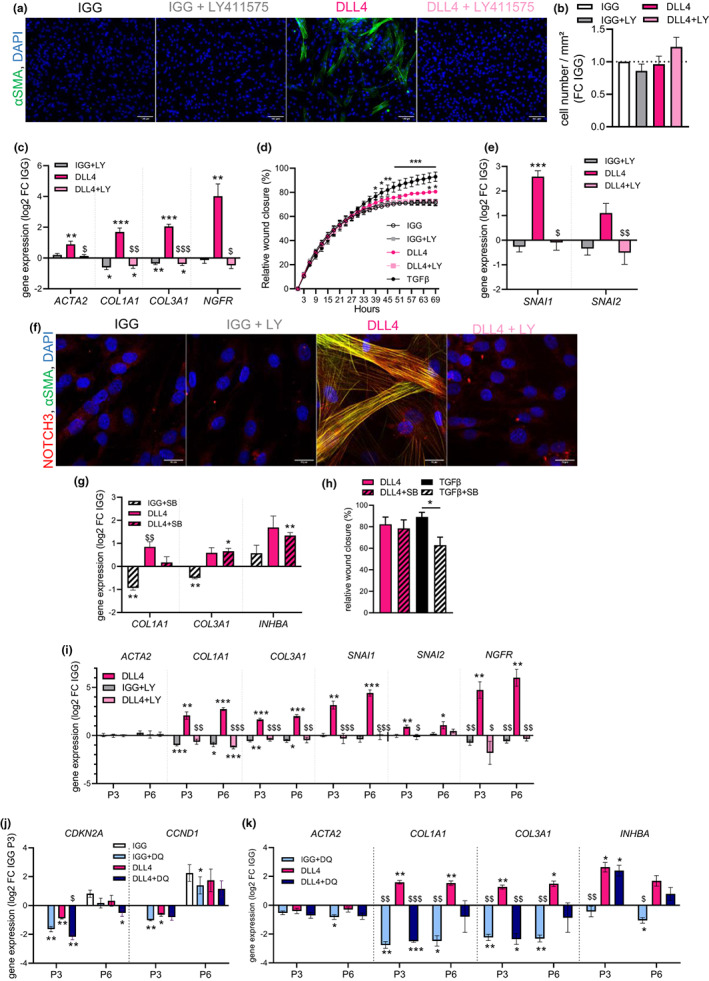
NOTCH‐mediated promotion of myofibrogenesis is not dependent of the TGFβ pathway. (a) Representative photomicrographs of αSMA (green) and DAPI (blue) stainings in immuno‐selected ScAT P1 progenitor cells seeded on immobilized IGG control (white) or NOTCH ligand DLL4 (pink) and cultured in basal medium (without TGFβ1) supplemented or not with gamma‐secretase inhibitor LY411575 (LY: IGG gray; DLL4 light pink) during 4 days. (scale bar = 100 μm). (b) Quantification of cell number at day 4. Results are expressed as fold change over IGG, means ± SEM of *n* = 3 donors (one‐way ANOVA, Tukey's post‐test). (c) mRNA levels of myofibroblast markers (*ACTA2*, *COL1A1*, and *COL3A1)* determined at day 4 by RTqPCR. (d) Results are expressed as log2 fold change over IGG, means ± SEM of *n* = 7–11 donors two‐way ANOVA, Dunnett's post‐test, *p < 0.05, **p < 0.01, ***p < 0.001 compared with IGG, $*p* < 0.05, $$*p* < 0.01, $$$*p* < 0.001 compared with DLL4). (d) Relative wound closure expressed as percentage of area over time of progenitor cells seeded on immobilized IGG control or DLL4, treated or not with gamma‐secretase inhibitor LY411575, or in the presence of TGFβ1 (black), and subjected to scratch assay. Images were captured every 3 h during 69 h. The scratch area at time point 0 was set to 0. Values are means ± SEM of *n* = 3 donors, two‐way ANOVA, Dunnett's post‐test, *p < 0.05, **p < 0.01, ***p < 0.001 (from time points 45–69) compared with IGG. (e) mRNA levels of epithelial‐mesenchymal transition genes *SNAI1* and *SNAI2* determined at day 4 by RTqPCR. Results are expressed as log2 fold change over IGG, means ± SEM of *n* = 7 donors (two‐way ANOVA, Dunnett's post‐test, ***p < 0.001 compared with IGG, $*p* < 0.05, $$*p* < 0.01 compared with DLL4). (f) Representative photomicrographs of NOTCH3 (red), DAPI (blue) and αSMA (green) stainings in progenitor cells seeded on IGG or DLL4 ligand with and without LY411575 at day 4 (scale bar = 20 μm). (g) mRNA levels of myofibroblast markers (*COL1A1*, *COL3A1*, *and INHBA*) in immuno‐selected P1 progenitor cells seeded on IGG (white) or NOTCH ligand DLL4 (pink) and cultured in basal medium supplemented or not with TGFβ receptor inhibitor SB431542 (IGG + SB hatched white, DLL4 + SB hatched pink) during 4 days, determined by RTqPCR. Results are expressed as log2 fold change over IGG, means ± sem of *n* = 4 donors (two‐way ANOVA, Tukey's post‐test, *p < 0.05, **p < 0.01 compared with IGG, $$*p* < 0.01 compared with DLL4). (h) Relative wound closure (expressed as percentage of area) 72 h after scratch wound of progenitor cells seeded on DLL4 or treated with TGFβ1 (black) in the presence or not of SB431542 (TGFβ + SB hatched black). Values are mean ± SEM of *n* = 4 donors (one‐way ANOVA and Sidak's post‐test). (i) mRNA levels of myofibroblast markers (*ACTA2*, *COL1A1*, and *COL3A1*), epithelial‐mesenchymal transition (*SNAI1* and *SNAI2*) and premyofibroblast marker (*NGFR*) in immuno‐selected ScAT P3 and P6 progenitor cells seeded on IGG control (white) or DLL4 (pink) and cultured in basal medium supplemented or not with gamma‐secretase inhibitor LY411575 (LY: IGG gray; DLL4 light pink) during 4 days determined by RTqPCR. Results are expressed as log2 fold change over IGG, means ± SEM of *n* = 7 donors (two‐way ANOVA, Tukey's post‐test, *p < 0.05, **p < 0.01, ***p < 0.001 compared with IGG, $*p* < 0.05, $$*p* < 0.01, $$$*p* < 0.001 compared with DLL4). (j) mRNA levels of senescence markers (*CDKN2A* and *CCND1*) in immuno‐selected ScAT P3 and P6 progenitor cells seeded on IGG control (white) or DLL4 (pink) and cultured in basal medium supplemented or not with dasatinib and quercetin (DQ; IGG + DQ light blue; DLL4 + DQ dark blue) during 4 days, determined by RTqPCR. Results are expressed as log2 fold change over P3 IGG, means ± SEM of *n* = 4 donors (two‐way ANOVA, Tukey's post‐test, *p < 0.05, **p < 0.01 compared with IGG, $*p* < 0.05 compared with DLL4). (k) mRNA levels of myofibroblast markers (*ACTA2*, *COL1A1*, *COL3A1*, and *INHBA*) determined at day 4 by RTqPCR. Results are expressed as log2 fold change over IGG, means ± SEM of *n* = 4 donors (two‐way ANOVA, Tukey's post‐test, *p 0.05, **p < 0.01, ***p < 0.001 compared with IGG, $*p* < 0.05, $$*p* < 0.01, $$$*p* < 0.001 compared with DLL4).

To further characterize the cell‐intrinsic impact of Notch activation in myofibrogenesis, immuno‐selected progenitor cells (P1) were cultured in the presence or absence of DLL4 and LY411575, without TGFβ1, during 4 days. Since JAG1 had at best a slight effect on myofibrogenesis, only the DLL4 ligand was used. Notch activation was sufficient to induce myofibroblast differentiation, as demonstrated by an increase in the number of αSMA‐positive cells and the induction of myofibroblast genes (*ACTA2*, *COL1A1*, and *COL3A1*) by day 4 (Figure 5a,b). In parallel, the expression of *NGFR,* a marker for the premyofibroblast subset (Esteve et al., 2019), was increased within 4 days (Figure 5c). Notch‐induced myofibrogenesis was completely abolished in the presence of the γ‐secretase inhibitor LY411575 (Figure 5a–c), indicating a requirement for NOTCH receptor cleavage. Neither DLL4 nor LY411575 affected the cell number (Figure 5b). In addition, wound closure assays clearly demonstrated that the stimulation of Notch signaling by DLL4 increased the migratory capacity of progenitor cells compared with the IGG control, but to a lesser extent than TGFβ treatment (Figure 5d). Notch activation increased EMT‐inducing transcription factors *SNAI1* and *SNAI2* (Figure 5e) in an LY411575 sensitive manner. Immunostainings revealed a cytoplasmic fiber localization of NOTCH3, which coincided with αSMA labeling, at day 4 only in DLL4‐differentiated progenitor cells (Figure 5f). Treatment with TGFβ receptor inhibitor SB431542 did not impact the DLL4‐induced upregulation of myofibroblast mRNA markers nor the DLL4‐mediated cell migration although it partially inhibited, as expected, the one stimulated by TGFβ (Figure 5g,h).C3H/HeOuJ mice, a strain known to develop AT fibrosis in diet‐induced obesity (Vila et al., 2014), were fed a high‐fat diet for up to 6 weeks, and the visceral AT was used in RTqPCR for myofibrogenic‐related genes *Acta2*, *Col1a1*, *Col3a1,* and *Eln*. While positive correlations were observed between *Notch3* expression and the panel of fibrosis markers, no correlations were found with *Notch1* mRNA levels (Figure S5a,b). In addition, a weak correlation was observed between *Notch3* and *Cdkn2a* levels but not with *Cdkn1a* or *Ccnd1*. Immuno‐selected ScAT progenitor cells were submitted to serial passaging and cultured at P3 and P6 for 4 days on DLL4 under basal conditions or in the presence of LY411575. Notch‐induced myofibrogenesis was maintained at P3 and P6, as shown by an increased expression in myofibroblast genes (*COL1A1*, *COL3A1*, and *INHBA*), EMT‐inducing transcription factors *SNAI1 and SNAI2* and premyofibroblast marker *NGFR* (Figure 5I). All effects of DLL4 were abrogated by the γ‐secretase inhibitor LY41575. Treatments of P3 and P6 progenitors with senolytics (dasatinib and quercetin) downregulated the expression of both *CDKN2A* and *CCND1*, as expected, with a stronger impact in P3 compared with P6 progenitors in the presence or not of DLL4 (Figure 5j). In parallel, senolytics repressed the basal expression of myofibrogenic‐related genes including *COL1A1* and *COL3A1* at P3 and P6 and the ones of *INHBA* and *ACTA2* at P6, as well as the upregulated expression of *COL1A* and *COL3A1* promoted by Notch signaling at P3 (Figure 5k).

### 3.6 Native premyofibroblasts accumulate in human VsAT with high levels of senescence and are the target of the NOTCH pathway

**FIGURE 6:**
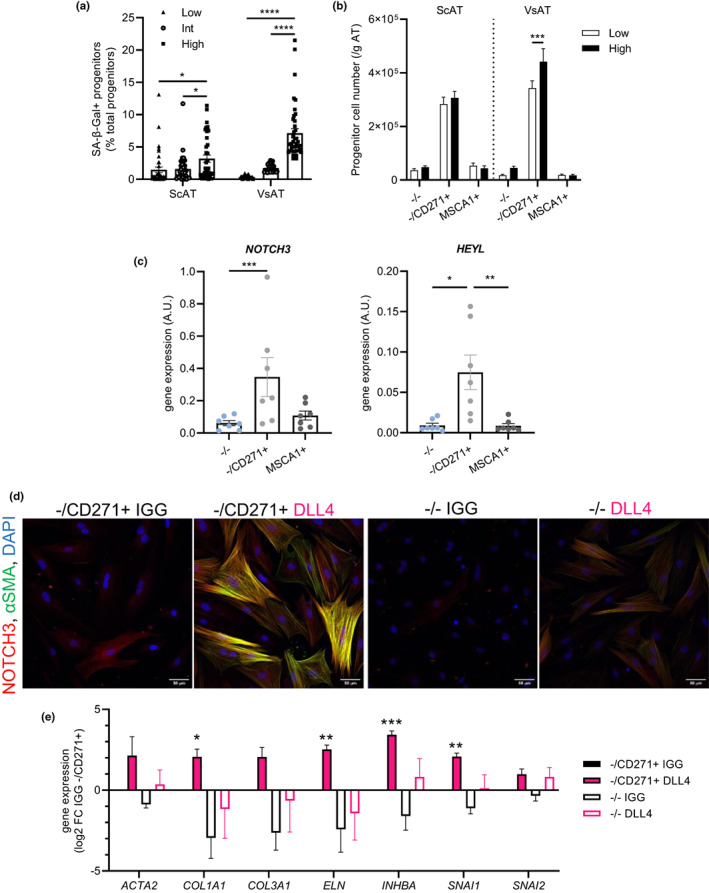
Native −/CD271^+^ premyofibroblasts accumulate in VsAT with senescence and are the target of the NOTCH3 pathway. (a) Percentage of SA‐β‐gal+ progenitors in ScAT and VsAT when donors were partitioned into tertiles by low, intermediate (int), or high SA‐β‐gal+ percentage in VsAT. Values represent means ± SEM of *n* = 38 donors in low, *n* = 39 in int and *n* = 39 in high VsAT senescence groups (two‐way ANOVA and Tukey's post‐test). (b) Number of progenitor cell subset per gram of ScAT and VsAT from patients in the low (white) and high (black) VsAT senescent tertiles from Figure 1e. Results are expressed as means ± SEM of *n* = 38 low and *n* = 39 high donors per group (two‐way ANOVA, Tukey's post‐test). (c) mRNA levels of *NOTCH3* and *HEYL* in freshly isolated progenitor cell subsets (MSCA1^−^/CD271^−^ (−/−, blue), MSCA1‐/CD271^+^ (−/CD271^+^, NGFR^+^, light gray) premyofibroblasts and MSCA1^+^ (ALPL^+^, dark gray) preadipocytes) immuno‐selected from ScAT. Results are expressed as means ± SEM of *n* = 7 donors (one‐way ANOVA, Dunn's post‐test). (d) Representative photomicrographs of NOTCH3 (red), αSMA (green) and DAPI (blue) stainings P1 in −/CD271^+^ and −/− progenitor subsets immuno‐selected from ScAT, seeded on IGG control or NOTCH ligand DLL4, and cultured in basal medium during 4 days (scale bar = 50 μm). (e) mRNA levels of myofibroblast markers (*ACTA2*, *COL1A1*, *COL3A1*, *ELN*, and *INHBA*) and epithelial‐mesenchymal transition markers (*SNAI1* and *SNAI2*) in −/CD271^+^ and −/− progenitor subsets seeded on IGG control (−/CD271^+^ black, −/− white) or NOTCH ligand DLL4 (−/CD271^+^ pink, −/− pink outline) determined at day 4 by RTqPCR. Results are expressed as log2 fold change over IGG −/CD271^+^ cells, means ± SEM of *n* = 5 independent experiments (two‐way ANOVA, Dunnett's post‐test, *p < 0.05, **p < 0.01, ***p < 0.001 compared with IGG

Obese patients were grouped in tertiles of low, intermediate, and high VsAT progenitor senescence. The group of patients with the highest percentage of SA‐β‐gal+ progenitors in VsAT had also a higher percentage of SA‐β‐gal+ progenitors in ScAT (Figure 6a). Among the clinical and anthropometrical characteristics and in agreement with the multivariate analysis (Figure 1e), the history of obesity reflected by the patient's BMI at the age of 20 was determinant of the degree of senescence in VsAT (Table 2). Age was also statistically significant between low and high senescence groups but with younger AT donors in the high senescence group (Table 2). Considering the two groups of patients with low and high percentage of VsAT senescent progenitors, we analyzed the progenitor subsets: MSCA1‐/CD271‐ (−/−) progenitors, MSCA1^−^/CD271+ (−/CD271^+^) premyofibroblasts, and MSCA1^+^ preadipocytes by flow cytometry. While the number of progenitor subsets was not different in ScAT between the two groups, there was a marked accumulation of −/CD271^+^ premyofibroblasts in VsAT from the high senescence group (Figure 6b). The adipocyte diameter distribution exhibited no differences in ScAT regardless of the degree of senescence; however, we found a reduction in the percentage of 20 μm small adipocytes in VsAT from the high senescence group (Figure S6). Finally, the three progenitor subsets were immuno‐selected from ScAT. RTqPCR analyses revealed higher expression levels of both *NOTCH3* and the NOTCH target gene *HEYL* in the −/CD271^+^ subset compared with −/− and MSCA1^+^ progenitor subsets (Figure 6c). Immuno‐selected −/CD271^+^ premyofibroblasts and −/− progenitors were seeded on DLL4 under basal conditions for 4 days (Figure 6e). NOTCH3 activation induced myofibroblast differentiation of −/CD271^+^ premyofibroblasts only, as shown by an increase in αSMA‐positive cells and myofibroblast gene expression (*COL1A1*, *ELN*, and *INHBA*), together with increased expression of the EMT‐inducing transcription factor *SNAI1* (Figure 6d,e). No effects were observed with the −/− progenitor subset.

**TABLE 2:** Clinical and anthropometric parameters according to VsAT progenitor senescence

	VsAT progenitor senescence								
	Low		Int		High				
Number of patients	38		39		39				
Ratio male/female	0.12		0.08		0.11				
Diabetic (%)	18.5		18.0		15.5				
	Mean	SD	Mean	SD	Mean	SD	Adj P low versus Int	Adj P low versus High	Adj P Int versus High
Glucose (mmol/L)	5.8	1.5	5.9	1.5	5.8	1.2	ns	ns	ns
HbA1c (%)	6.0	1.1	6.0	0.8	5.9	0.8	ns	ns	ns
Triglycerides (mmol/L)	1.5	0.9	1.3	0.7	1.3	0.6	ns	ns	ns
Cholesterol (mmol/L)	5.3	0.9	5.1	0.8	5.2	1.0	ns	ns	ns
HDL (mmol/L)	1.2	0.3	1.2	0.3	1.1	0.2	ns	ns	ns
LDL (mmol/L)	3.5	0.8	3.3	0.8	3.4	0.9	ns	ns	ns
SGOT/ASAT (IU/L)	24.1	12.8	24.7	10.8	23.6	10.2	ns	ns	ns
SGPT/ALAT (IU/L)	35.6	22.9	36.3	19.3	38.2	22.5	ns	ns	ns
HOMA‐IR	6.1	4.3	4.8	2.8	5.4	3.9	ns	ns	ns
Waist‐to‐hip ratio	0.98	0.12	0.93	0.13	0.95	0.12	ns	ns	ns
Age (years)	43.39	9.84	41.60	10.26	39.41	9.83	ns	0.0351	ns
BMI (kg/m^2^) at 20 years	28.22	6.96	30.02	7.46	32.27	8.40	ns	0.0307	ns
Actual BMI (kg/m^2^)	43.23	4.56	44.47	6.06	45.06	7.05	ns	ns	ns

*Note*: Statistical analyses were performed using two‐way ANOVA followed by Tukey's multiple comparisons test (ns nonsignificant).

## 4 DISCUSSION

There has been an increasing amount of evidence, implicating that adipose tissue (AT) senescence plays a role in both obesity‐ and age‐induced processes responsible for metabolic abnormalities. We show that SA‐β‐Gal‐positive cells accumulate in both ScAT and VsAT of obese subjects by colorimetric imaging approaches, as described previously (Conley et al., 2020; Gustafson et al., 2019; Rouault et al., 2021). Differences in whole tissue SA‐β‐Gal staining have been reported between ScAT and VsAT of obese patients (Rouault et al., 2021). Unsupervised analysis of flow cytometry datasets allows the identification of SA‐β‐Gal+ cells in the SVC, revealing that macrophages represent the largest proportion, followed by progenitors and endothelial cells. SA‐β‐Gal+ macrophages have been previously reported in AT, but their senescent phenotype remains debated (Hall et al., 2017). Additional experiments are needed to further characterize the SA‐β‐Gal+ macrophage population in human AT. They are probably located within the SA‐β‐Gal+ crown‐like structures we observed surrounding human adipocytes in this study, also described in mouse AT (Rabhi et al., 2022). While in ScAT, the majority of SA‐β‐Gal+ stromal cells are macrophages, progenitors and macrophages are equally represented in VsAT. No difference in the absolute number of SA‐β‐Gal+ macrophages is found between fat depots. The endothelial cell cluster, which does contain SA‐β‐Gal+ cells, consists of a limited number of cells, due to the small biopsy size, and was not analyzed further. Endothelial senescence has been shown to be sufficient to induce adipocyte dysfunction and metabolic disorders in a mouse model (Barinda et al., 2020). We previously reported higher endothelial senescence in VsAT from obese individuals compared with ScAT and with age in ScAT (Briot et al., 2018; Villaret et al., 2010). Finally, SA‐β‐Gal+ progenitors are both modulated by AT location in terms of their proportion and number, with higher amounts found in VsAT. Progenitors are key cellular components for fat depot repair, physiological turnover, and expansion under energy excess. Although adipocyte turnover is considered to be a slow process in adults (Spalding et al., 2008), chronic renewal pressure exerted on progenitor cells is higher in obese versus lean individuals due to the greater number of adipocytes. In the present study, multivariable unbiased analysis highlighted the association between senescence parameters and obesity, more marked in VsAT compared with ScAT. Moreover, the group of patients with the highest proportion of senescent progenitors in VsAT is characterized by an early obesity trajectory reflected by an increased BMI at the age of 20 years old. It is therefore tempting to speculate that high VsAT progenitor senescence is a consequence of the chronic replicative pressure exerted by a longer history of obesity. The proliferative rate of AT progenitor cells is distinct according to fat depot location, with less proliferation in visceral compared with subcutaneous AT (Tchkonia et al., 2006). The present data suggest that the depot‐specific differences in senescence may be an additional determinant of the progenitor proliferation rate. Combined transcriptomics and proteomics datasets, obtained from both native and in vitro replicative senescent immuno‐selected progenitors and comparison with other available SASP datasets, highlight common core SASP factors in both ScAT and VsAT including INHBA as well as fat depot‐ and replicative senescence‐specific SASP factors. In particular, GREM1 and SFRP4, endogenous antagonists of the BMP and WNT pathways, respectively, are markers of the VsAT secretome and elevated with replicative senescence. SFRP4 and GREM1 are not only emerging adipokines with higher expression in VsAT (Hedjazifar et al., 2020) but also contained within published senescent gene sets (Gustafson et al., 2019) (https://senequest.net/). In addition to finding BMP and WNT antagonists in the SASP, our study highlights the importance of the Notch developmental pathway in the senescence‐associated intrinsic phenotype. Increased NOTCH3 expression has already been reported during replicative senescence induced by telomere shortening in human cell lines (Cui et al., 2013). Our present data demonstrate that NOTCH3 expression also increased with senescence in human AT progenitors. This increase is specific for NOTCH3 since the mRNA levels of *NOTCH1* are not impacted and specific to replicative senescence compared with oxidative or DNA damage stress. The canonical Notch target genes *HES1* and *HEYL* were not modulated with cell passaging, suggesting that replicative senescence is not associated with a sustained activation of Notch‐dependent pathway without precluding a pulsatile activation (Nandagopal et al., 2018) and/or oscillatory changes in the levels of these genes with short mRNA half‐lives (Kobayashi & Kageyama, 2014). Developmental pathways modulate progenitor fate during self‐renewal and differentiation. While BMP and WNT‐dependent pathways are potent pro‐adipogenic stimuli in AT, the net impact of NOTCH signaling on adipogenesis, in particular NOTCH1, is controversial (Shan et al., 2017). As an antidevelopmental process, senescence is more often associated with impaired stem cell/progenitor differentiation. Senescent AT stromal cells from aged/obese mice and humans exhibit a decreased adipogenic potential (Le Pelletier et al., 2021; Xu, Palmer, et al., 2015), while a targeted reduction in senescence enhances adipogenesis (Xu, Palmer, et al., 2015). Depending on tissue context and cell type, senescence may also promote the myofibrogenic differentiation, such as in wounded skin (Demaria et al., 2014) or inhibit it through a modulation of Notch/TGFβ axis (Lopez‐Antona et al., 2022). Our data demonstrate that Notch activation has a major impact in the determination of AT progenitor fate since it impairs white and brite adipogenesis while promoting myofibrogenesis. Both NOTCH ligands, DLL4 and JAG1, did not trigger similar effects, with weak to no impact of JAG1. Although additional experiments will be needed to clearly define the underlying molecular mechanism, distinct ligands can activate, through the same Notch receptor, different target genes and cell fate by defining distinct Notch receptor activation dynamics, that is, sustained or pulsatile (Nandagopal et al., 2018). Interestingly, the dynamic fluctuation of NOTCH1 activity associated with oncogene‐induced senescence has been involved in the balance between distinct SASP promoting a TGFβ rich secretome (Hoare et al., 2016). The present study clearly showed that the promotion of myofibrogenesis by Notch activity was observed even in the presence of exogenously added TGFβ, strongly suggesting additive distinct mechanisms. In agreement, the pharmacological inhibition of the TGFβ pathway did not alter the Notch‐dependent activation of progenitor migration and myofibrogenesis. Notch activity in nonsenescent and in replicative senescent progenitors promoted the expression of SASP‐related factors including SFRP4 and INHBA. In oncogene‐induced senescence, the SASP induces a secondary senescence in surrounding cells that is mediated by Notch signaling (Teo et al., 2019). Whether a similar impact is at play in the AT progenitors remains to be studied but is unlikely since Notch activation was associated with an inhibition rather than a stimulation of the senescent markers *CDKN2A* and *CCDN1*. NOTCH3 has already been involved in fibrosis (Ramachandran et al., 2019). Since positive correlations between *NOTCH3* (but not with *NOTCH1*) and myofibrogenic‐related gene expression were observed in human and mouse AT, it is strongly suggested that NOTCH3 is also the active Notch receptor in AT myofibrogenesis although gene editing approaches will permit to clearly state about the molecular identity of the Notch receptor. We show that NOTCH3 protein, upon activation, localizes on fibers, a staining pattern that can be found within the Human Protein Atlas. While further investigation is required to conclude that the change in protein subcellular localization from membranes to fibers is responsible for cytoskeletal remodeling of myofibroblasts, it is associated with increased migratory capacity and expression of EMT transcription factors, *SNAI1* and *SNAI2*. Our data show that the NOTCH3‐dependent pathway is enriched in the −/CD271^+^ premyofibroblasts compared with the other cell subsets and sufficient to induce their differentiation. Additionally, the group of patients with the highest progenitor senescence in VsAT is characterized by higher −/CD271^+^ premyofibroblast accumulation, supporting the hypothesis that NOTCH activity contributes to the depot‐specific enrichment of −/CD271^+^ subsets that we previously reported (Esteve et al., 2019). Our findings also suggest that it will be worthwhile to investigate the role of NOTCH3 in wound healing, especially in light of a recent study describing that insufficient induction of AT senescence after injury is a pathological mechanism of diabetic wound healing (Kita et al., 2022).

As a consequence of the defect in adipocyte renewal, the accumulation of SA‐β‐Gal+ cells in mouse and human ScAT has been associated with adipocyte hypertrophy (Gustafson et al., 2019; Xu, Palmer, et al., 2015) and has a negative impact on systemic metabolism. In agreement with a recent study (Ishaq et al., 2022), we did not observe differences in adipocyte hypertrophy nor in systemic metabolic parameters in the patients grouped according to VsAT progenitor senescence. One cannot exclude that other cell types, including adipocytes, endothelial cells, and/or macrophages may provide a stronger contribution than progenitors alone in the link between SA‐β‐Gal+ cell accumulation and adipocyte hypertrophy and/or metabolic impairment associated with obesity. Whether such a mechanism is also involved in aging remains to be established, especially since uncontrolled fibrosis is a common hallmark of aged tissues; referred to as “fibroageing” (Selman & Pardo, 2021). We did not observe positive association between the accumulation of senescent progenitors and age but rather the inverse association; however, our cohort was not designed for the study of chronological aging but rather for the study of obesity‐accelerated aging. Finally, since in vitro cell expansion is required for mesenchymal stromal cell‐based regenerative therapy, NOTCH3 may represent an interesting molecular actor to monitor and target replicative senescence.

## AUTHOR CONTRIBUTIONS

N.B., A.Br., J‐C.G., and A.Bo. designed the experiments. N.B., V.J., D.E., A.R., C.B., P.V., J.F., L.M., P.D., E.M., C.C., and J.G. performed experiments and collected the data. A.Z.G. isolated the progenitor subsets with cell sorter for in vitro studies. C.D., M.G., C.P., Y.V., M.D., and J.S.I. were involved in generating and analysis of mass spectrometry and transcriptomic data. S.L. collected AT and patient information from the SENADIP cohort. J‐L.G. collected AT from dermolipectomies. N.B., A.Br., J.G., J‐C.G., and A.Bo. were involved in the guidance and concept developments of the study. N.B. and A.Bo. wrote the paper. All authors carefully read and reviewed the final version of the paper.

## CONFLICT OF INTEREST

The authors declare no competing interests.

## Supporting information


Supinfo

## Data Availability

All RNAseq datasets generated and used in this study will be deposited and available from the NCBI Gene Expression Omnibus (GEO) portal after the acceptance of the manuscript.
