# Supplementary material for: Notch activation shifts the fate decision of senescent progenitors toward myofibrogenesis in human adipose tissue
Source: Aging Cell. 2023 Jan 8;22(3):e13776. doi: 10.1111/acel.13776 (PMC10014050; doi:10.1111/acel.13776)
Supplement: Supplementary file 1 — Supinfo [file ACEL-22-e13776-s001.pdf]

Supplementary Figure 1

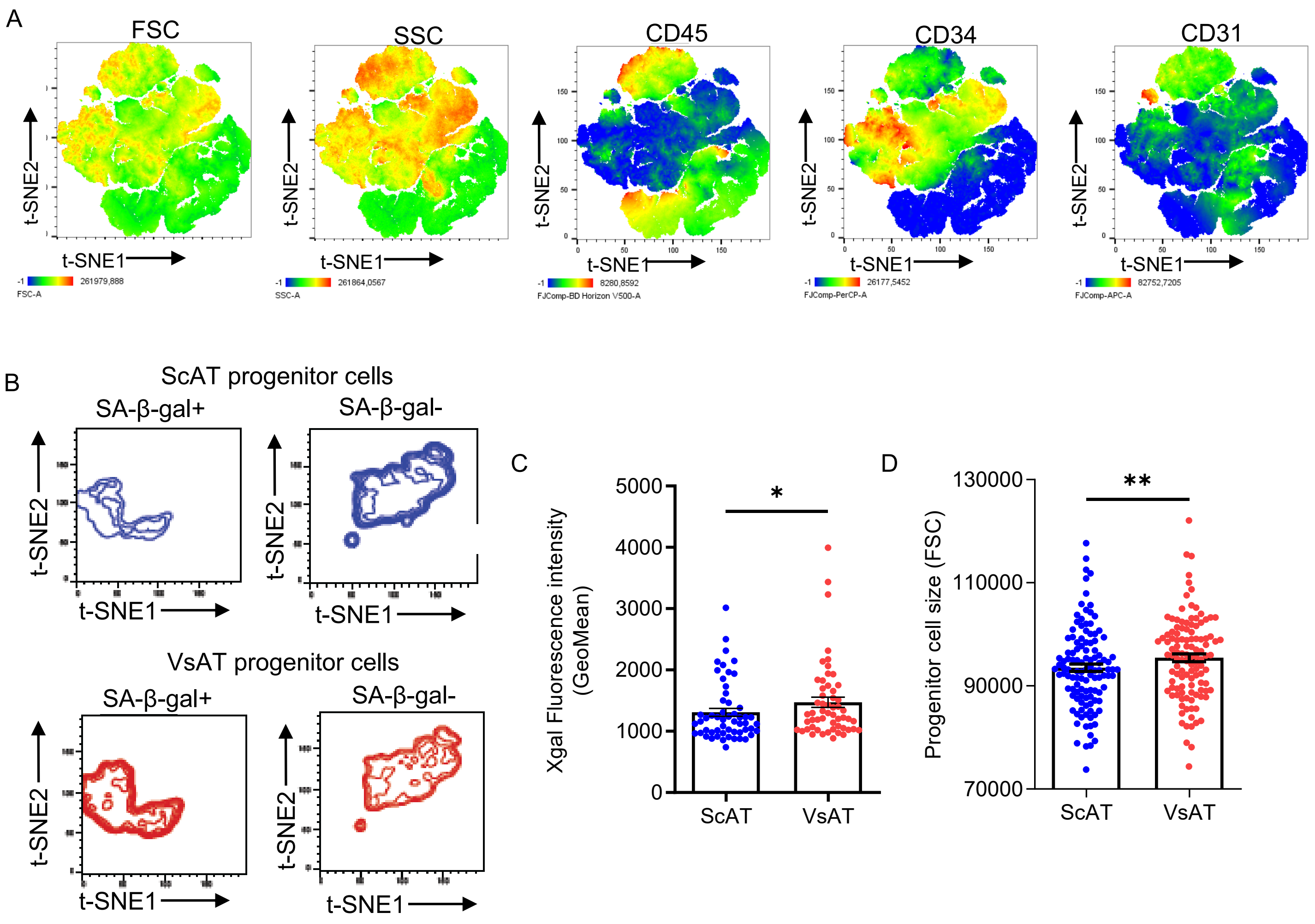

Supplementary Figure 1: **Characterization of ScAT and VsAT progenitor senescence by flow cytometry.**
A) Paired ScAT and VsAT stroma-vascular cells from 55 subjects were analysed by flow cytometry. tSNE of flow cytometry data after downsampling and concatenation of the FSC (Forward Scatter), SSC (Side Scatter), CD45, CD34 and CD31 fluorescence intensities. B) Density plot of the SA-β-gal+ and SA-β-gal- ScAT and VsAT progenitor clusters. C) Geometric mean of the SA-β-gal fluorescence intensity in SA-β-gal+ progenitor cells of ScAT and VsAT from 55 subjects. D) Progenitor cell size determined with the FSC in ScAT and VsAT by supervised analysis of the SA-β-gal+ progenitor cells. Results are expressed as means +/- sem.

Supplementary Figure 2

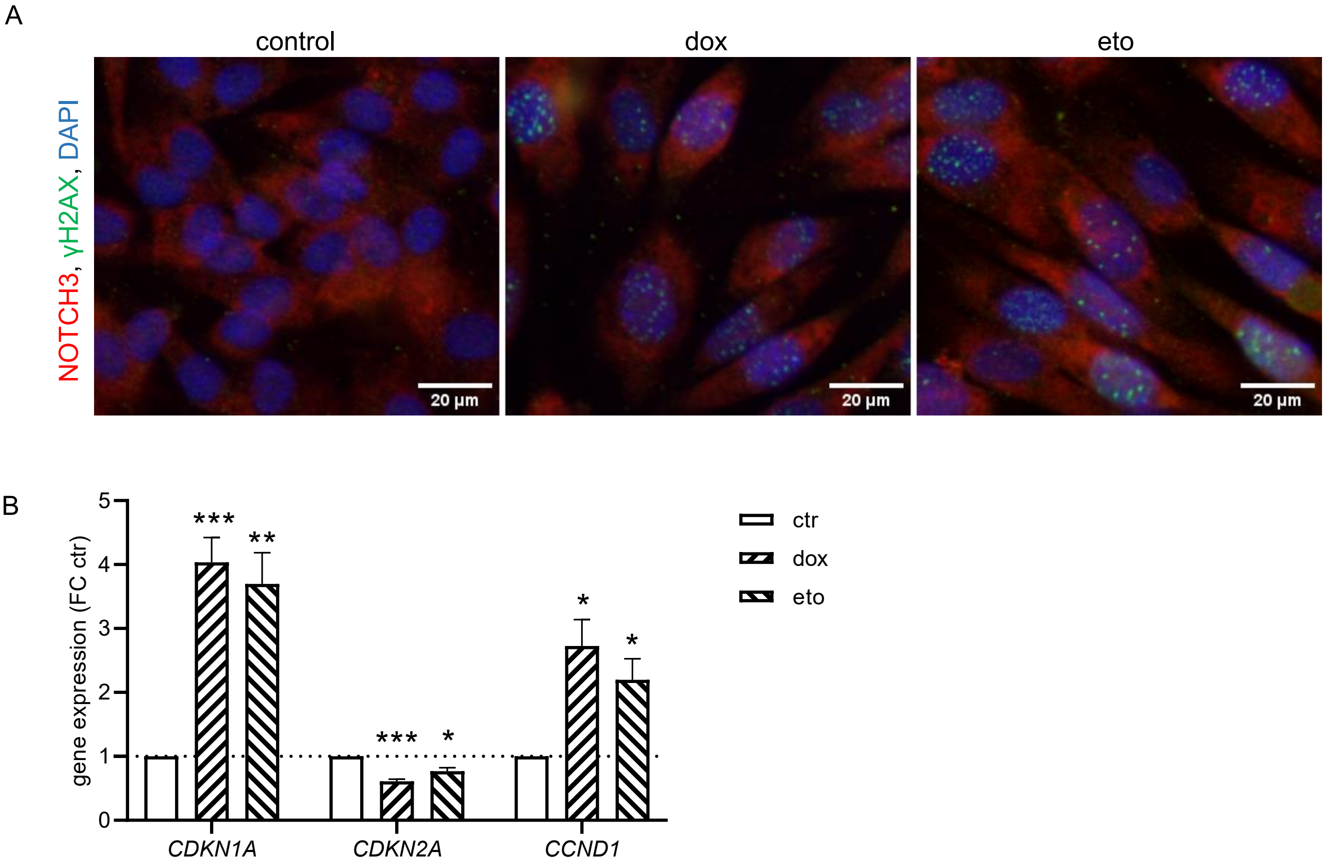

Supplementary Figure 2: **DNA damage-induced senescence in ScAT progenitors.**

A) Representative photomicrographs of NOTCH3 (red), γH2AX (green) and DAPI (blue) stainings in progenitor cells treated with doxorubicin (dox) or etoposide (eto) or not (control) during 72h (scale bar = 20µm).

B) *CDKN1A, CDKN2A and CCND1* mRNA levels in progenitor cells after 72 hours of doxorubicin (dox) or etoposide (eto) treatment determined by RTqPCR. Results are expressed as fold change over control cells, means +/- sem of n=3 donors (two-way ANOVA, Dunnett's post-test, * p<0,05, ** p<0,01, *** p<0,001 compared to control).

Supplementary Figure 3

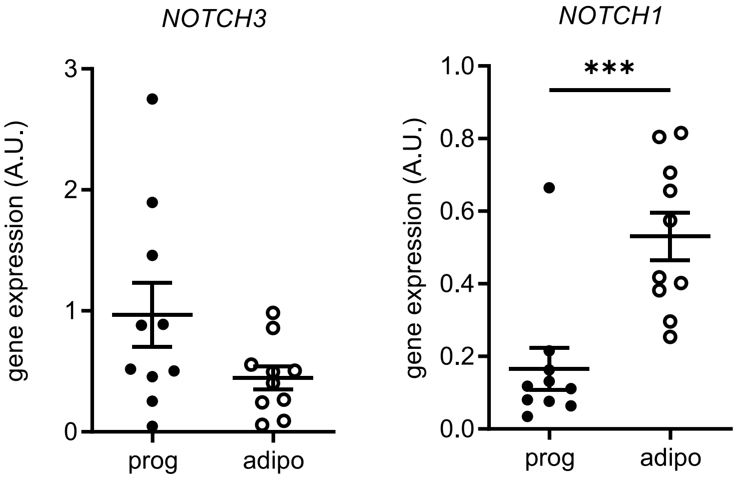

Supplementary Figure 3: **Distinct expression of *NOTCH1* and *NOTCH3* according to obesity and cell type.** *NOTCH3* and *NOTCH1* mRNA levels in isolated progenitor cells (prog) and mature adipocytes (adipo) determined by RTqPCR. Values are means +/- sem of n=10 donors.

Supplementary Figure 4

A

ScAT

	NOTCH3	*ACTA2*	*COL1A1*	*COL3A1*	*INHBA*	*GREM1*	*SFRP4*	*ADIPOQ*	*GPD1*	*PLIN1*	*PPARG*	*NOTCH1*	*SNAI1*	*SNAI2*	*TGFB1*
NOTCH3	1.00	0.65	0.42	0.38	0.44	0.22	0.32	-0.57	-0.48	-0.41	-0.46	0.19	0.06	0.06	0.32
ACTA2	0.65	1.00	0.37	0.24	0.67	0.16	0.39	-0.57	-0.54	-0.48	-0.56	-0.11	0.02	0.05	0.22
COL1A1	0.42	0.37	1.00	0.87	0.39	0.36	0.77	-0.46	-0.44	-0.41	-0.63	-0.32	-0.30	0.22	0.49
COL3A1	0.38	0.24	0.87	1.00	0.20	0.23	0.72	-0.29	-0.25	-0.23	-0.43	-0.23	-0.27	0.22	0.39
INHBA	0.44	0.67	0.39	0.20	1.00	0.24	0.45	-0.58	-0.61	-0.59	-0.64	-0.21	-0.14	0.14	0.24
GREM1	0.22	0.16	0.36	0.23	0.24	1.00	0.24	-0.32	-0.28	-0.28	-0.36	-0.05	-0.10	0.02	0.35
SFRP4	0.32	0.39	0.77	0.72	0.45	0.24	1.00	-0.38	-0.38	-0.34	-0.59	-0.32	-0.29	0.15	0.32
ADIPOQ	-0.57	-0.57	-0.46	-0.29	-0.58	-0.32	-0.38	1.00	0.91	0.89	0.79	0.20	0.11	-0.22	-0.45
GPD1	-0.48	-0.54	-0.44	-0.25	-0.61	-0.28	-0.38	0.91	1.00	0.92	0.76	0.26	0.12	-0.24	-0.38
PLIN1	-0.41	-0.48	-0.41	-0.23	-0.59	-0.28	-0.34	0.89	0.92	1.00	0.74	0.28	0.11	-0.28	-0.45
PPARG	-0.46	-0.56	-0.63	-0.43	-0.64	-0.36	-0.59	0.79	0.76	0.74	1.00	0.34	0.21	-0.14	-0.44
NOTCH1	0.19	-0.11	-0.32	-0.23	-0.21	-0.05	-0.32	0.20	0.26	0.28	0.34	1.00	0.35	-0.11	-0.19
SNAI1	0.06	0.02	-0.30	-0.27	-0.14	-0.10	-0.29	0.11	0.12	0.11	0.21	0.35	1.00	-0.20	-0.06
SNAI2	0.06	0.05	0.22	0.22	0.14	0.02	0.15	-0.22	-0.24	-0.28	-0.14	-0.11	-0.20	1.00	0.24
TGFB1	0.32	0.22	0.49	0.39	0.24	0.35	0.32	-0.45	-0.38	-0.45	-0.44	-0.19	-0.06	0.24	1.00

1.0 0.5 0 -0.5 -1.0

VsAT

B

	NOTCH3	*ACTA2*	*COL1A1*	*COL3A1*	*INHBA*	*GREM1*	*SFRP4*	*ADIPOQ*	*GPD1*	*PLIN1*	*PPARG*	*NOTCH1*	*SNAI1*	*SNAI2*	*TGFB1*
NOTCH3	1.00	0.50	0.22	0.28	0.18	0.08	-0.02	-0.26	-0.23	-0.19	-0.13	0.28	0.03	0.13	0.19
ACTA2	0.50	1.00	0.07	0.10	0.51	0.06	0.12	-0.22	-0.21	-0.21	-0.20	-0.07	-0.05	0.03	0.01
COL1A1	0.22	0.07	1.00	0.91	0.13	0.25	0.53	-0.35	-0.31	-0.31	-0.50	-0.34	-0.34	0.13	0.24
COL3A1	0.28	0.10	0.91	1.00	0.09	0.23	0.47	-0.26	-0.22	-0.22	-0.37	-0.27	-0.32	0.10	0.23
INHBA	0.18	0.51	0.13	0.09	1.00	0.12	0.23	-0.17	-0.20	-0.23	-0.24	-0.03	0.15	0.19	0.10
GREM1	0.08	0.06	0.25	0.23	0.12	1.00	0.43	-0.41	-0.31	-0.25	-0.37	-0.22	-0.33	-0.21	0.20
SFRP4	-0.02	0.12	0.53	0.47	0.23	0.43	1.00	-0.38	-0.29	-0.26	-0.50	-0.45	-0.48	-0.23	0.01
ADIPOQ	-0.26	-0.22	-0.35	-0.26	-0.17	-0.41	-0.38	1.00	0.93	0.88	0.80	0.41	0.33	-0.01	-0.29
GPD1	-0.23	-0.21	-0.31	-0.22	-0.20	-0.31	-0.29	0.93	1.00	0.93	0.79	0.39	0.24	-0.11	-0.31
PLIN1	-0.19	-0.21	-0.31	-0.22	-0.23	-0.25	-0.26	0.88	0.93	1.00	0.75	0.37	0.16	-0.18	-0.36
PPARG	-0.13	-0.20	-0.50	-0.37	-0.24	-0.37	-0.50	0.80	0.79	0.75	1.00	0.56	0.42		-0.23
NOTCH1	0.28	-0.07	-0.34	-0.27	-0.03	-0.22	-0.45	0.41	0.39	0.37	0.56	1.00	0.50	0.19	0.02
SNAI1	0.03	-0.05	-0.34	-0.32	0.15	-0.33	-0.48	0.33	0.24	0.16	0.42	0.50	1.00	0.38	0.20
SNAI2	0.13	0.03	0.13	0.10	0.19	-0.21	-0.23	-0.01	-0.11	-0.18		0.19	0.38	1.00	0.32
TGFB1	0.19	0.01	0.24	0.23	0.10	0.20	0.01	-0.29	-0.31	-0.36	-0.23	0.02	0.20	0.32	1.00

1.0 0.5 0 -0.5 -1.0

Supplementary Figure 4: **Correlation between *NOTCH3* and *NOTCH1* with fibrosis-, adipogenic- and TGFβ-related genes in ScAT and VsAT.**
Correlation matrix showing the Spearman r between *NOTCH3, NOTCH1* and fibrosis-related (*ACTA2*, *COL1A1*, *COL3A1*), SASP-related (*INHBA*, *GREM1*, *SFRP4*), adipogenic-related (*ADIPOQ*, *GPD1*, *PLIN1*, *PPARG*) and TGFβ-related genes (*SNAI1*, *SNAI2*, *TGFβ1*) transcript levels in A) ScAT and B) VsAT from the GTEx RNA-seq database performed on whole AT samples. ScAT n=663, VsAT n=541, Spearman correlation test.

Supplementary Figure 5

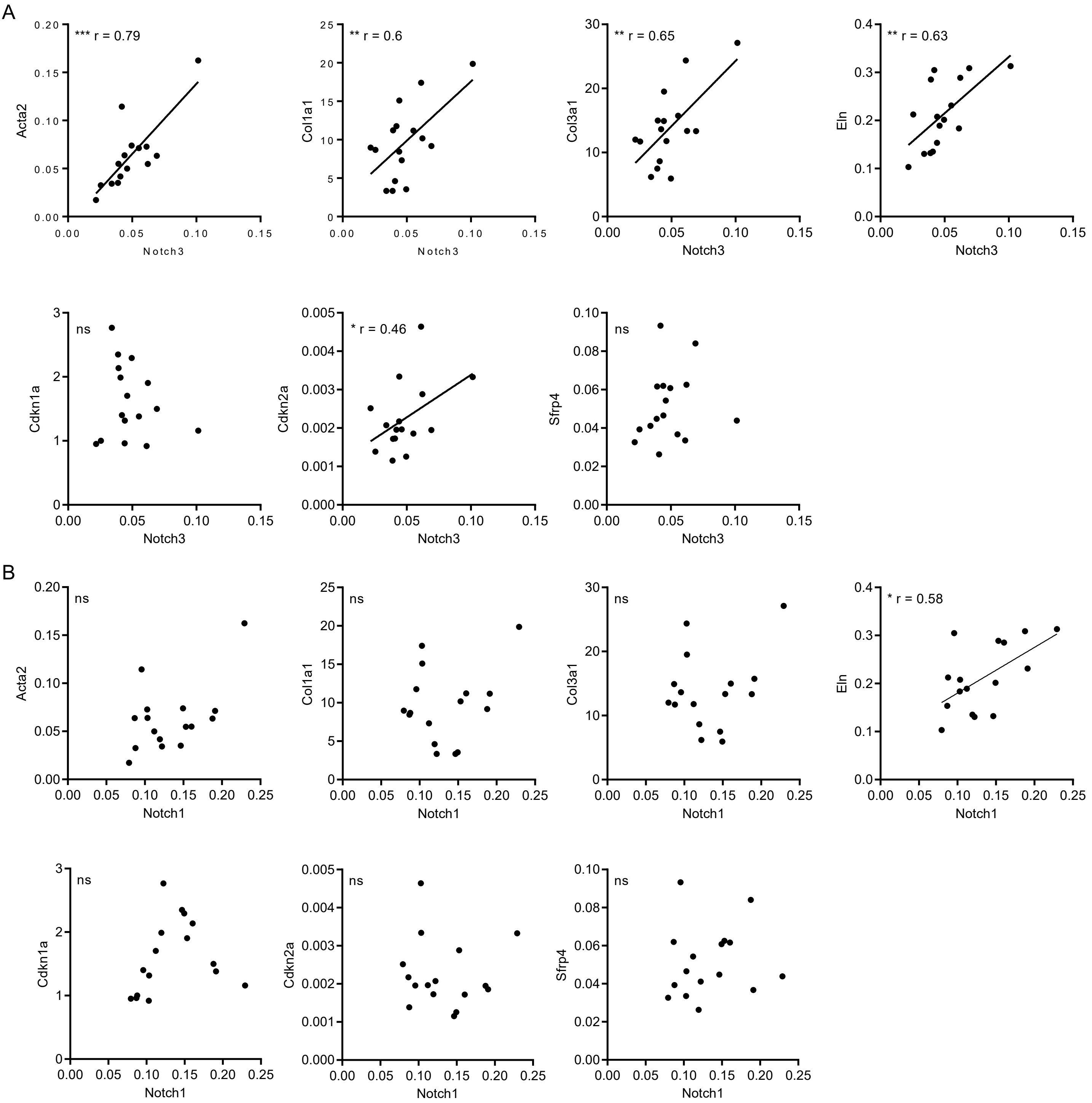

Supplementary Figure 5: ***Notch3* gene expression is positively correlated with fibrosis-related genes in VsAT stroma-vascular cells of C3H/HeOuJ mice.**

Correlations between A) *Notch3* or B) *Notch1* and fibrosis-related genes (*Acta2*, *Col1a1*, *Col3a1*, *Eln*) and senescence-related genes (*Cdkn1a*, *Cdkn2a*, *Sfrp4*) in stroma-vascular fraction cells isolated from perigonadal AT of mice fed 0, 2, 4 and 6 weeks with 45% high fat diet, determined by RTqPCR. n=16, Pearson correlation test.

Supplementary Figure 6

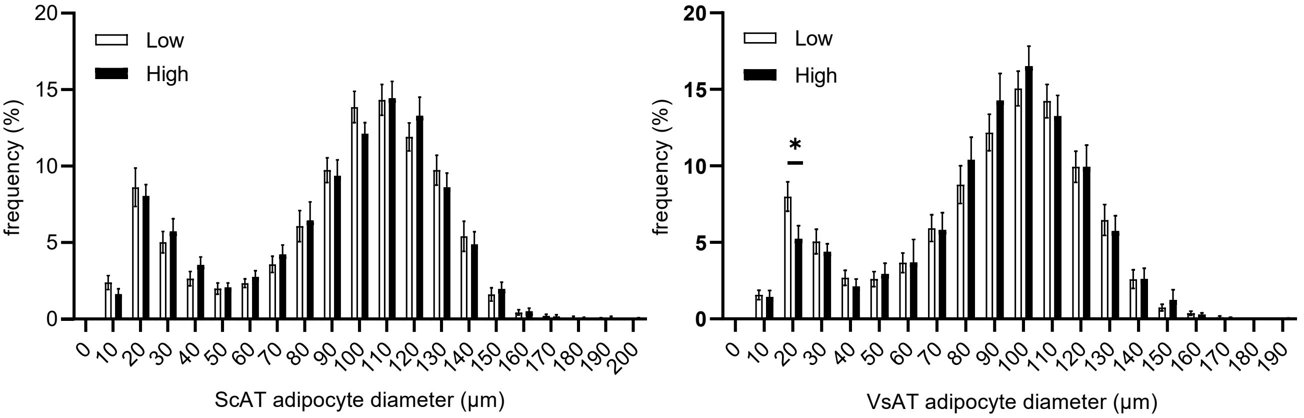

Supplementary Figure 6: **ScAT and VsAT adipocyte diameter in low and high senescence groups.** Distribution of mature adipocyte diameter in ScAT (left panel) and VsAT (right panel) in low and high VsAT progenitor senescence groups. Attention to the VsAT 20 µm diameter position where the only significant difference was observed between the low and high tertiles. Results are expressed as means +/- sem of n=30 low and n=24 high donors per group (two-way ANOVA, Tukey’s post-test).

Supplementary Table 1: Human and murine Assay on Demand (Thermofisher)

Human target gene	AOD
ACTA2	HS00909449m1
ADIPOQ	Hs00605917-m1
CCND1	Hs00277039-m1
CDKN1A	Hs00355782-m1
CDKN2A	Hs00923894-m1
CITED1	Hs00918445-g1
COL1A1	Hs00164004-m1
COL3A1	Hs00943809-m1
CYCS	Hs01588973-m1
DIO2	Hs00988260-m1
ELN	Hs00355783-m1
ELOVL3	Hs00537016-m1
GPD1	Hs00193386-m1
GREM1	Hs01879841-s1
HES1	Hs00172878-m1
HEYL	Hs01113778-m1
INHBA	Hs00170103-m1
NGFR	Hs00182120-m1
NOTCH1	Hs00413187-m1
NOTCH2	Hs01050702-m1
NOTCH3	Hs01128537-m1
PLIN1	Hs00160173-m1
PPARGC1A	Hs01016719-m1
PPARGC1b	Hs00991677-m1
PPARG2	Hs01115510-m1
PPIB	Hs00168719-m1
SFRP4	Hs00180066-m1
SNAI1	Hs00195591-m1
SNAI2	Hs00161904-m1
TFAM	Hs01082775-m1
UCP1	Hs00222453-m1

Murine target gene	AOD
Acta2	Mm01546133-m1
Cdkn1a	Mm00432448-m1
Cdkn2a	Mm00494449-m1
Col1a1	Mm00801666-g1
Col3a1	Mm00802300-m1
Eln	Mm00514670-m1
Notch1	Mm00627185-m1
Notch3	Mm01345646-m1
Sfrp4	Mm00840101-m1
